# Tenovin-1, a Selective SIRT1/2 Inhibitor, Attenuates High-fat Diet-induced Hepatic Fibrosis via Inhibition of HSC Activation in ZDF Rats

**DOI:** 10.7150/ijbs.97304

**Published:** 2024-06-11

**Authors:** Swati Sharma, Sreevarsha Gali, Amit Kundu, Jae Hyeon Park, Jae-Sung Kim, Hyung Sik Kim

**Affiliations:** 1 School of Pharmacy, Sungkyunkwan University, 2066, Seobu-ro, Jangan-gu, Suwon 26419, Republic of Korea.; 2 Department of Pharmacology, GITAM School of Pharmacy, GITAM (Deemed to be University), Rushikonda, Visakhapatnam-530045, Andhra Pradesh, India.; 3 Department of Surgery, Washington University in St. Louis, St. Louis, MO, 63110, USA.

**Keywords:** High-fat diet, Hepatic Fibrosis, Tenovin-1, Oxidative Stress, Inflammation, Hepatic Stellate Cells

## Abstract

Type 2 diabetes mellitus (T2DM) increases the risk of non-alcoholic fatty liver disease (NAFLD) progression to advanced stages, especially upon high-fat diet (HFD). HFD-induced hepatic fibrosis can be marked by oxidative stress, inflammation, and activation of hepatic stellate cells. Sirtuin 1/2 (SIRT1/2), NAD-dependent class III histone deacetylases, are involved in attenuation of fibrosis. In our conducted research, TGF-β1-activated LX-2 cells, free fatty acid (FFA)-treated simultaneous co-culture (SCC) cells, and HFD-induced hepatic fibrosis in Zucker diabetic fatty (ZDF) rats, a widely used animal model in the study of metabolic syndromes, were used to evaluate the protective effect of Tenovin-1, a SIRT1/2 inhibitor. ZDF rats were divided into chow diet, HFD, and HFD + Tenovin-1 groups. Tenovin-1 reduced hepatic damage, inhibited inflammatory cell infiltration, micro/ macro-vesicular steatosis and prevented collagen deposition HFD-fed rats. Tenovin-1 reduced serum biochemical parameters, triglyceride (TG) and malondialdehyde (MDA) levels but increased glutathione, catalase, and superoxide dismutase levels. Tenovin-1 mitigated proinflammatory cytokines IL-6, IL-1β, TNFα and fibrosis biomarkers in HFD rats, TGF-β1-activated LX-2 and FFA treated SCC cells. Additionally, Tenovin-1 suppressed SIRT1/2 expression and inhibited JNK-1 and STAT3 phosphorylation in HFD rats and FFA-treated SCC cells. In conclusion, Tenovin-1 attenuates hepatic fibrosis by stimulating antioxidants and inhibiting inflammatory cytokines under HFD conditions in diabetic rats.

## Introduction

The development of non-alcoholic fatty liver disease (NAFLD) is closely associated with type 2 diabetes mellitus (T2DM), obesity, and dyslipidemia. Patients with T2DM have been found to exhibit advanced forms of NAFLD, including hepatic fibrosis, regardless of whether they are obese or not 1. This complicated association between T2DM and NAFLD involves a bidirectional link between their pathophysiological mechanisms. Evidence suggests that T2DM contributes to the development and progression of NAFLD 2. It is considered a risk factor for the rapid progression of NAFLD into nonalcoholic steatohepatitis (NASH), fibrosis, and cirrhosis.

In patients with diabetes, NAFLD progression is influenced by multiple factors, including insulin resistance, hyperinsulinemia, and prediabetes. Other factors such as obesity, HFD, and lack of physical activity worsen the risk of NAFLD in these patients 3. T2DM increases insulin resistance associated lipid accumulation in the liver followed by inflammation, leading to steatohepatitis, which can further advance to hepatic fibrosis upon progression 4. Upon high-calorie diet consumption, an increase in the flux of free fatty acids (FFAs) causes an increase in the accumulation of intracellular lipids within hepatocytes, which in turn impairs FFA oxidation and amasses toxic lipid metabolites and reactive oxygen species (ROS) in the liver 5,6.

Insulin resistance-mediated oxidative stress with decreased antioxidant defense systems has been suggested to be one of the leading causes of hepatic fibrosis associated with T2DM. Many pathological conditions in fibrotic diseases are characterized by the accumulation of intracellular ROS and the formation of profibrotic myofibroblasts depends on intracellular ROS. Sustained elevation of ROS can induce the progenitor cells to attain a fibrogenic phenotype 7. Additionally, various compounds with antioxidant properties have been used to attenuate or protect against fatty liver diseases and fibrosis 8. Furthermore, ROS-mediated liver injury also involves NF-*κ*B activation to produce pro-inflammatory cytokines such as IL-1*β,* TNF*α*, and IL-6. Studies have shown an increase in hepatic inflammation following HFD, with an increase in inflammatory cytokine levels 9. These cytokines, along with other inflammatory mediators from the fibrotic tissues promote HSC activation and trans-differentiation into extracellular matrix (ECM)-secreting myofibroblasts. These HSCs are highly fibrogenic, proliferative, migratory, contractile, and pro-inflammatory thus, contributing to a fibrotic liver. Although a few therapies have shown improvement of hepatic fibrosis in T2DM, no effective antifibrotic drugs have been approved for either diabetic or non-diabetic patients 10.

Sirtuins (SIRTs), class III NAD+-dependent histone deacetylases, comprise seven genes (*SIRT1-7*), as identified in mammalian cells. SIRTs remove acetyl groups from histones and transcription factors and promote gene transcription 11. The SIRT family has been associated with progression of liver fibrosis. SIRT1 is a histone deacetylase known to be involved in fatty acid synthesis, oxidation, and adipogenesis. It also plays a valuable role in mitigating NAFLD 12. SIRT2 inhibition, on the other hand, has been shown to alleviate organ fibrosis, but its role in liver fibrosis remains unclear. However, some reports have suggested that SIRT2 inhibition represses fibrogenic genes in HSCs to prevent hepatic fibrosis 13. The studies involving dual SIRT1/2 inhibitors have also shown some affirmative results in various fibrotic diseases 14,15. In studies involving hepatic fibrosis, inhibitors like suramin exhibited anti-fibrotic properties, whereas AGK2 was observed to inhibit alpha-smooth muscle actin (α-SMA), an important marker of fibrosis 16,17.

Tenovin-1 is a novel compound that inhibits SIRT1/2 by inhibiting its deacetylase activity. The structure consists of a thiourea moiety in the center, flanked by an acetamide group and a tert-butylbenzamide group on the two sides 18. The tert-butylbenzamide group provides lipophilicity to the molecule, which contributes to its SIRT1/2 inhibition activity. Tenovin-1 affects SIRT1 and SIRT2 substrate acetylation in mammalian cells. Inhibition of this activity by Tenovin-1 showed promising outcomes in melanoma, wherein it exhibited better suppression of cell viability and proliferation in comparison to other sirtuin inhibitors such as Sirtinol and Ex-527 19. Tenovin-1 can mimic the effect of byproduct of sirtuin reaction, nicotinamide, a physiological sirtuin inhibitor. In a study, Tenovin-1 induced cellular senescence and impaired wound-healing activity in rat primary astrocytes corresponding to its SIRT1/2 inhibitory activity which may have effects on brain aging and neurodegenerative diseases 20. Another study reported that Tenovin-1 is a promising drug against flavivirus infection which was associated to its SIRT2 inhibition property. Recently, Tenovin-1 has also been proposed as a potential treatment option for COVID-19 infection 21. The reno-protective effect of Tenovin-1 has also been studied in rats with diabetic nephropathy, which showed its positive antifibrotic effects 22. However, the effect of Tenovin-1 on NAFLD and hepatic fibrosis remains unclear.

In the present study, we hypothesized that a SIRT1/2 inhibitor, due to its SIRT1/2 deacetylase inhibition activity, can attenuate HSC activation and HFD-induced hepatic fibrosis in diabetic rats. It was established that this decrease in the fibrotic levels were due to a reduction in oxidative stress, inflammatory mediators, and hepatocyte apoptosis which could lead to the inactivation of HSC. Based on previous research, the anti-inflammatory and anti-oxidative activities of SIRT1/2 inhibitor were attributed to the reduction in the phosphorylation of JNK and STAT3 signaling. For investigating this hypothesis, we explored the role of a recently discovered SIRT1/2 inhibitor, Tenovin-1, in oxidative stress, inflammation, and fibrosis associated with high-fat diet (HFD)-induced hepatic fibrosis. The effects of Tenovin-1 were evaluated in Zucker diabetic fatty (ZDF) rats, a widely used model of diabetes and obesity. We also evaluated its effects on HSC activation in an *in vitro* co-culture system to understand the molecular mechanisms involved in the functional role of SIRT1/2 inhibition in liver fibrogenesis. Our findings demonstrate an improved understanding towards the role of SIRT1/2 inhibition in liver fibrosis with or without diabetes and validates Tenovin-1 as a potential option for its treatment.

## Materials and Methods

### Reagents and antibodies

Tenovin-1(N-[[[4-(acetylamino) phenyl] amino] thioxomethyl-4-(1,1-dimethyl ethyl)] benzamide) was purchased from Cayman Chemicals (Ann Arbor, MI, USA). Polyclonal antibodies (pAb) against α-SMA (ab5694), fibronectin (ab2413), IL-6 (ab6672), IL-1β (ab9722), TGF-β1 (ab215715), BAX (ab32503), E-cadherin (ab40772), and JNK-1 (ab47337) were acquired from Abcam (Cambridge, UK). pAb against vimentin (#5741s), collagen-type I alpha 1 (COL1A1) (#72026), SIRT1(#8469), p-STAT3(#9145), STAT3 (#9139S), TNF-α (#8184S), cleaved caspase 3 (#9661), BCL-2 (#15071), p-c-JUN (#9164), and c-JUN (#9165) were purchased from Cell Signaling Technology (Danvers, MA, USA), whereas SIRT2 (sc-28298), p-JNK (sc-6254), and β-actin (sc-47778) were procured from Santa Cruz Biotechnology (Santa Cruz, CA, USA). The bicinchoninic acid (BCA) kit was purchased from Thermo Fisher Scientific (Waltham, MA, USA). Secondary antibodies conjugated with Horseradish peroxidase (HRP) were obtained from Novus Biologicals (Centennial, CO, USA) and GeneTex, Inc. (Irvine, CA, USA).

### Experimental model

Male ZDF rats (5 weeks old) weighing 250 ± 25 g were obtained from Central Lab Animal Inc. (Seoul, Korea). The rats were housed in a pathogen-free environment with a constant temperature of 25 ± 0.5 °C and a relative humidity of 53-57%. The rats were kept in a pathogen-free room and the temperature of the room was maintained at 25 ± 0.5 °C and relative humidity of 53-57%. For acclimatization, the animals were housed in a 12-hour light/dark cycle for seven days, with unlimited access to food and water. All animal experiments received an approval from the Animal Ethics Committee (SKKUIACUC2018-10-32-1). The experimental procedures involving the use of animals adhered to the ethical code of conduct. The HFD comprised 60% fat, carbohydrates, proteins, vitamins, and minerals. The high-fat diet was purchased from Research Diets, Inc. (New Brunswick, NJ, USA).

**Figure 1:**
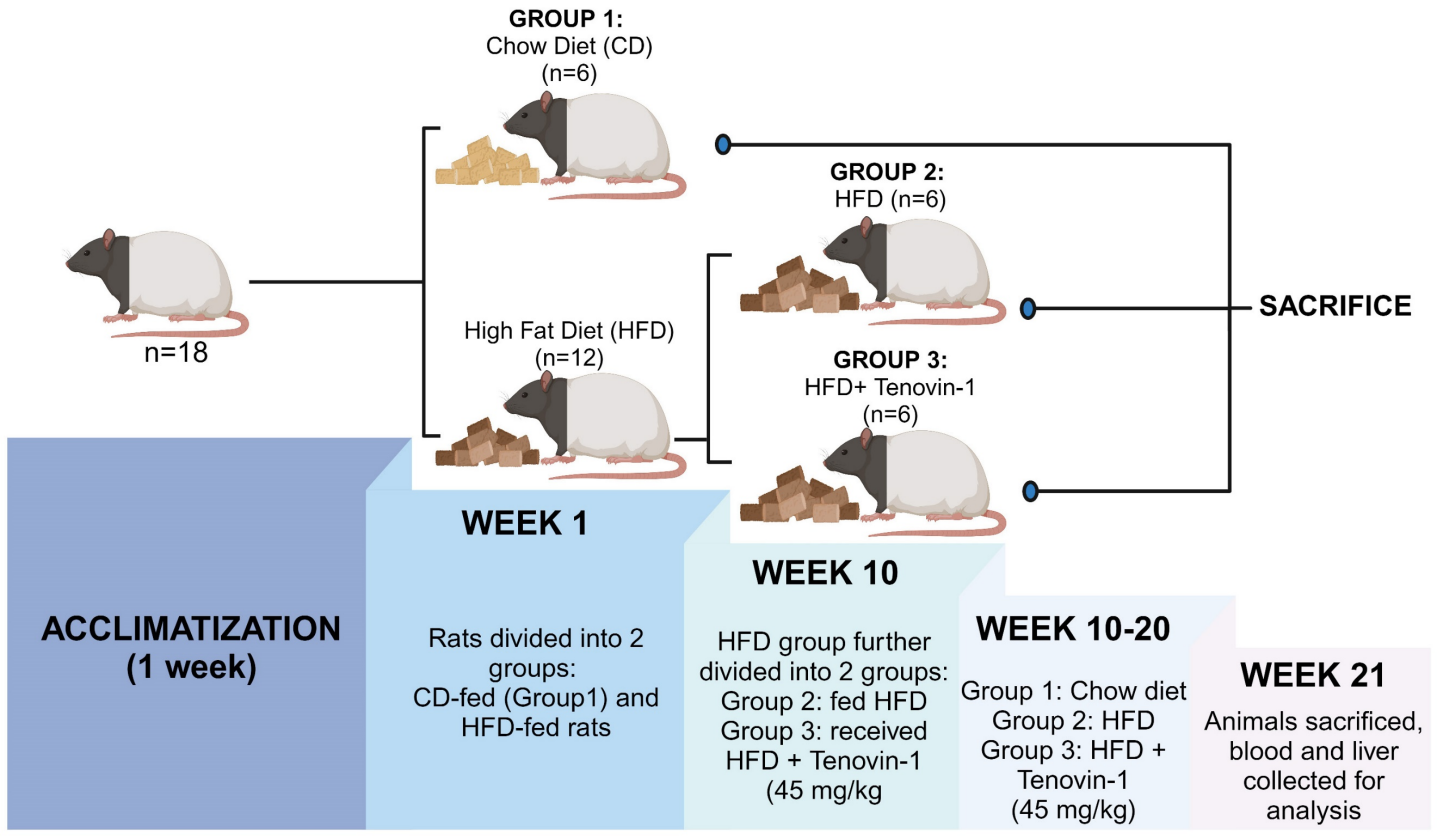
**Experimental design.** Following a week of acclimatization, 18 rats were randomly divided in two groups with six rats in each: the CD group was fed with a chow diet. The Experimental group was fed with a HFD (n = 12). After 10 weeks, the experimental group was further divided into two groups (n = 6 each): HFD (n = 6) and HFD + Tenovin-1(n = 6) for 10 weeks. CD, chow diet; HFD, high-fat diet. After sacrifice, blood and liver samples were collected and stored for further analysis. (Created with BioRender.com)

In the 1^st^ week, 18 ZDF rats were randomly divided into two groups: Chow diet (CD) group (n=6) fed with a normal chow diet of 4.5% fat, and the experimental group (n=12) fed with HFD for the induction of hepatic fibrosis. After 10 weeks, the experimental group was further divided into two groups of six rats each (n=6): HFD group fed with HFD diet and HFD + Tenovin-1 group (45 mg/kg body weight, intraperitonially) 19 receiving HFD followed by Tenovin-1 administration for the next 10 weeks. The CD and HFD groups received normal saline as a vehicle. Eight weeks after the initiation of the experiment, the rats were weighed twice weekly until sacrifice. In the 21^st^ week, the rats were euthanized using isoflurane. Blood was collected from the hepatic portal vein and the serum was separated from blood by centrifugation at 2000 × *g* for 10 min. The serum obtained, was stored at -20 °C for further experiments. The rat livers were removed, weighed, placed in normal saline to remove blood, and then stored at -80 °C **(Figure 1)**.

### Evaluation of histopathology, steatosis, and fibrosis

Haematoxylin and Eosin (H&E) staining measures morphological changes, steatosis, fibrosis, and inflammation, whereas Masson's trichrome (MT) and Sirius red staining measure the degree of collagen-1 deposition in the liver section. Liver tissues were fixed using 4% neutral-buffered paraformaldehyde, were dehydrated, followed by embedment in paraffin after slicing into 3-5 µm sections. The tissue sections were rehydrated with xylene, ethanol and stained with the H&E/Masson's Trichrome/Picro-Sirius Red Stain (Abcam). For Oil Red O (ORO) staining, frozen liver tissues were cut into sections and stained with ORO solution to measure the triglyceride (TG) and lipid levels. Morphological changes, the degree of steatosis (red), fibrosis, and collagen deposition (blue/red) were attained using a K1-fluo microscope (Nanoscope Systems Inc., Daejeon, Korea) at 200x or 400x magnification.

### Analysis of serum biochemical parameters

Serum collected after sacrificing the animals was used to estimate biochemical parameters. Aspartate aminotransferase (AST), alanine aminotransferase (ALT), alkaline phosphatase (ALP), gamma-glutamyltransferase (GTP) were analyzed using a VetScan analyzer (Abaxis, Union City, CA, USA) in the serum samples whereas low density lipoprotein (LDL), high density lipoprotein (HDL), and TG levels whereas were measured using a UV-visible spectrophotometer-V-650 (JASCO Inc. Easton, MD, USA) at 505 nm. Total cholesterol (TC) was measured at 560 nm using a spectrophotometer.

### Assessment of oxidative stress parameters

Liver tissues (25-30 mg) were homogenized and centrifuged for 10 min at 1500 × *g*. After centrifugation, the supernatant was extracted to be used for the following assays. Malondialdehyde (MDA), a well-known biomarker of lipid peroxidation, was measured using a thiobarbituric acid reactive substance (TBARS) assay kit (Cayman Chemicals, Ann Arbor, MI, USA). The absorbance MDA level in the samples was measured at 540 nm and expressed as μM. A colorimetric superoxide dismutase (SOD) assay kit (Cayman Chemicals, Ann Arbor, MI, USA) was used to determine SOD levels according to the instructions of the manufacturer. Absorbance of SOD activity was measured at 450 nm and was expressed as U/ml. The peroxidase activity of catalase (CAT) was measured using a colorimetric CAT assay kit (Cayman Chemicals, Ann Arbor, MI, USA) according to the instructions of the manufacturer. CAT activity was measured at 540 nm using a microplate reader and was expressed as nmol/mg protein. Total glutathione (GSH) was measured using a glutathione (total) detection kit (Enzo Life Sciences, NY, USA) following the manufacturer's protocol. Total GSH was detected using a VetScan analyzer (Abaxis, Union City, CA, USA) and expressed as pmol/ml.

### Evaluation of inflammatory cytokines by ELISA

Pro-inflammatory and anti-inflammatory cytokine, interleukin (IL)-6, IL-10, IL-1β, and (tumor necrosis factor-α) TNF-α levels were assessed in liver tissue. Tissues were weighed (25-30 mg) and lysed in the lysis buffer, followed by centrifugation for 10 min at 2000 × *g*. The supernatants were used for cytokine estimation using ELISA kits (Abcam, Cambridge, MA, USA), according to the protocol of the manufacturer. The reagents were made in accordance with the manufacturer's instructions, and standard dilutions were prepared as required. A hundred microliters of each standard and samples were added to the wells in duplicates or triplicates and incubated at 20-25 °C with gentle shaking. Washing buffer was used to clean the wells. Cytokine-specific biotinylated antibodies were added, followed by 1-2 h of incubation, and shaking. After subsequent washing, the wells were incubated with HRP-streptavidin for 1 h at 20-25 °C with shaking. After another rinse, TMB substrate solution was added to the wells. The stop solution was added after 15 to 30 minutes, and a spectrophotometer was used to detect the optical density immediately at 450 nm. Cytokine levels were expressed as pg/ml or μg/ml protein.

### Western blot analysis

Protein extraction was carried out from the liver tissues and *in vitro* samples. Liver tissue (approximately 30 mg) and cells were homogenized in RIPA buffer. The lysates were centrifuged at 2000 × *g* for 10 min. Protein concentration in the supernatant was estimated using a bicinchoninic acid (BCA) assay. Samples were prepared in 5X sample buffer and loaded onto 8-15% sodium dodecyl sulfate-polyacrylamide gel electrophoresis (SDS-PAGE) for separation of the proteins via electrophoresis. The separated proteins were transferred onto nitrocellulose membranes. To block nonspecific binding, membrane proteins were incubated with 5% BSA or skim milk. The membranes were then incubated with specific primary antibodies (1:1000) at 4 °C overnight. The blots were washed and incubated with horseradish peroxidase-conjugated secondary antibodies (1:10000) for 1 h at room temperature. The chemiluminescence blots were enhanced using Immobilon Western HRP Substrate (Merck Millipore, Darmstadt, Germany), and the intensity of the bands was visualized using a WSE-6200H Luminograph II (Atto, Taito-ku, Tokyo, Japan) and quantified using ImageJ software (NIH, USA).

### TUNEL assay

A terminal deoxynucleotidyl transferase (TdT) dUTP Nick-End Labeling (TUNEL) assay was performed to evaluate DNA degradation during apoptosis in liver tissue samples. TUNEL apoptotic detection kit was used for the assay according to the instruction of the manufacturer. Briefly, paraffin embedded tissue sections were used for the assay. The sections were deparaffinized and rehydrated using xylene and ethanol. Permeabilization was done using 100 μL of Proteinase K for 20 min. After washing, endogenous peroxidase inactivation was done using 100 μL of methanol diluted H_2_O_2_ for 5 min and washed. Terminal Deoxynucleotidyl Transferase (TdT) equilibration buffer was added for 30 min for equilibration. Working TdT Labeling Reaction Mixture was applied, covered with coverslip, and incubated at room temperature for 1.5 h. Coverslip was removed, slides were washed with TBS and 100 μl of stop solution for 5 min was added. After wash, the tissue specimen was covered with blocking buffer for 10 min at room temperature. 100 μL of 1X Conjugate diluted in Blocking Buffer for 30 min was added followed by washing. This step was followed by addition of 100 μL of DAB solution for 15 min and rinsing with distilled H_2_O. Methyl Green was used as counter stain and added for 1-3 min. The tissues were dehydrated using in ethanol, xylene and a glass coverslip was mounted. TUNEL-positive hepatic cells were imaged using a K1-fluo microscope (Nanoscope Systems Inc., Daejeon, Korea) at 200× magnification.

### Cell line, culture, simultaneous co-culture system and treatments

The American Type Culture Collection (ATCC, Manassas, Virginia, USA) provided the immortalized human hepatic stellate cell line LX-2 and the human hepatocellular carcinoma cell line HepG2. After thawing, the cells were grown in Dulbecco's minimum essential medium (DMEM) supplemented with 10% fetal bovine serum (FBS), penicillin (100 U/mL), and streptomycin (100 g/mL) in a T-75 flask. The cells were incubated at 37 °C in a 5% CO_2_ humidified atmosphere. Upon 80-85% confluency, the cells were trypsinized with 0.05% trypsin and EDTA solution (0.53 mM). The cells were subcultured every 2 d. LX-2 cells were seeded for 24 h, exposed to recombinant Human TGF-β1 (240-B-002) obtained from R&D systems at 5 ng/ml concentration to induce HSC activation, and then treated with different concentrations of Tenovin-1. Dimethyl sulfoxide (DMSO; D8418, Sigma-Aldrich, St. Louis, MO, USA) was used as a control. For the simultaneous co-culture (SCC) system model, HepG2 and LX-2 cells were seeded at a ratio of 5:1 in a single plate in 1% FBS medium to avoid pre-activation of LX-2. After seeding for 24 h, the cells were exposed to FFA, a mixture of BSA-conjugated oleic acid (OA, O1008, Sigma-Aldrich) and palmitic acid (PA, P5585, Sigma-Aldrich, USA) in a ratio of 2:1 at 1 mM in high glucose media with 1% FBS for 24 h to mimic the fibrosis-like characteristics 23. The control group was treated with bovine serum albumin (BSA) (A8806, fatty acid-free; Sigma-Aldrich).

### Cell viability

Cell proliferation was evaluated using a WST-1 assay according to the instructions of the manufacturer. 96-well plates were used to seed the cells in 100 μL DMEM/well and incubated under 37 °C and 5% (v/v) CO_2_ for 24 h. Following incubation, cells were exposed to different doses of Tenovin-1 for 24 h. Treatment period was followed by adding 10 μL/well WST-1 reagent and incubation for 1 h. Absorbance was assessed using a VERSA MAX Microplate Reader (Molecular Devices Corp., CA, USA) at 450 nm.

### Wound healing assay

The LX-2 cells were seeded in 96-well plates. Cells were grown until they reached 90% confluence. Wounds were created in each well using an Essen wound maker (Essen Bioscience, MI, USA). The cells were washed two times using serum-free DMEM followed by treatment with TGFb1 (5 ng/ml) and Tenovin-1 at different concentrations. DMSO were treated the control group. Cell migration was observed, recorded, and quantified using Biotek Cytation 5 (Agilent, Santa Clara, CA, USA).

### Hydroxyproline level determination

The hydroxyproline content provides a direct measure of the amount of collagen. Following the manufacturer's instructions, hydroxyproline was determined using an assay kit (Abcam, Cambridge, MA, USA). Liver tissues were weighed (25-30 g) and homogenized in 10 mL of 5N HCl following an overnight incubation at 120 °C. The tissues were hydrolyzed during incubation. The product was filtered through a PVDF syringe filter (0.45 μm). The 100μL filtrate was added to 96-well plate, sealed, and incubated in a microplate incubator for 45 min at 65 °C. Absorbance was measured at 560 nm and the results were expressed as micrograms per milligram of protein.

### mRNA expression analysis

TRIzol reagent (Invitrogen, Carlsbad, CA, USA) was used to homogenize 30 g of liver for total RNA extraction. The RNA was quantified at 280 nm using a spectrophotometer (NanoDrop Technologies, Wilmington, DE, USA). Accordingly, cDNA was synthesized using a reverse transcription 5x PreMastermix (elpis biotech, Daejeon, Korea) in a PCR thermal cycler (TaKaRa PCR Thermal Cycler Dice Gradient, Shiga, Japan). cDNA was used as a template with SYBR Premix Ex TaqTM (Life Technologies) to perform qRT-PCR using Real-Time PCR System (LightCycler 96, Roche, Basel, Switzerland). The conditions were as follows: initial denaturation at 95 °C for 3 min followed by 30 cycles at 95 °C for 30 s, 50 °C for 30 s, and 68 °C for 30 s with final extension at 72 °C for 5 min. The primers used in the experiment were purchased from BIONEER (Bioneer Inc., Daejeon, Korea) and are listed in Table S1. β-Actin was used as the internal control.

### Immunohistochemistry, immunocytochemistry, and immunofluorescence

Paraffin-embedded tissue slides were deparaffinized followed by subsequent immersion in xylene, ethanol (100%, 95%, and 70%) for 5 min each for rehydration. Antigen retrieval in sodium citrate buffer (pH 6.0) was carried out at the sub-boiling temperature for 20 min. Endogenous peroxidase activity was inhibited by cooling tissue sections with hydrogen peroxide in methanol. Blocking was done using 10% normal goat serum for 1 h followed by incubation with primary antibody against SIRT1, SIRT2, α-SMA, collagen-1 alpha 1 (COL1A1), fibronectin, TGF-βR1, p-JNK, or p-STAT3 at 4 °C overnight. After 3 washings with 1XTBS, the tissues were incubated with horseradish peroxidase (HRP)-conjugated secondary antibodies for 1 h. For immunofluorescence, the tissues were incubated with DAPI for nuclear staining at 0.1-1 μg/ml) for 1 min, rinsed five times and mounted. For immunohistochemistry (IHC), tissues were incubated for 30 min with streptavidin-HRP conjugate at 20-25 °C and *DAB Chromogen solution* (3, 3′-diaminobenzidine) was added used to visualize the antibody-stained section. The tissues were counterstained with hematoxylin. Ammonium hydroxide was added as a bluing reagent for 1 min. The tissues were immersed in ethanol and xylene for dehydration and were mounted. Photomicrographs were obtained using a K1-fluo microscope (Nanoscope Systems Inc., Daejeon, Korea) at 200×.

For immunocytochemistry (ICC), confocal dishes were used to seed the cells, followed by for 24 h of treatment. Fixation of the cells was done using 4% paraformaldehyde and were permeabilized using TritoxX-100. The cells were blocked for 1 h using 1% bovine serum albumin (BSA) in PBST. Blocking was followed by incubation in primary antibody for another hour at 20-25 °C or 4 °C overnight. After decanting and washing the cells with PBS, they were exposed to a secondary antibody diluted in 1% BSA for 1 h of incubation. Counted staining was performed using DAPI (0.1-1 μg/ml). The cells were rinsed with PBS and photomicrographed under K1-fluo microscope (Nanoscope Systems Inc., Daejeon, Korea) at 200× magnification.

### Statistical analysis

All the data shown in the study have been expressed as mean ± standard deviation (SD) of six rats. Statistical significance was determined using one-way analysis of variance (ANOVA) method. Bonferroni's post hoc t-test was used for multiple comparisons. GraphPad prism software v5.0 (GraphPad, San Diego, CA, US) was used to conduct statistical analysis, and a *p* value < 0.05 indicated statistical significance.

## Results

### Tenovin-1 mitigates HFD-induced increase in body and liver weight along with histopathological changes in ZDF rats

**Figure 2:**
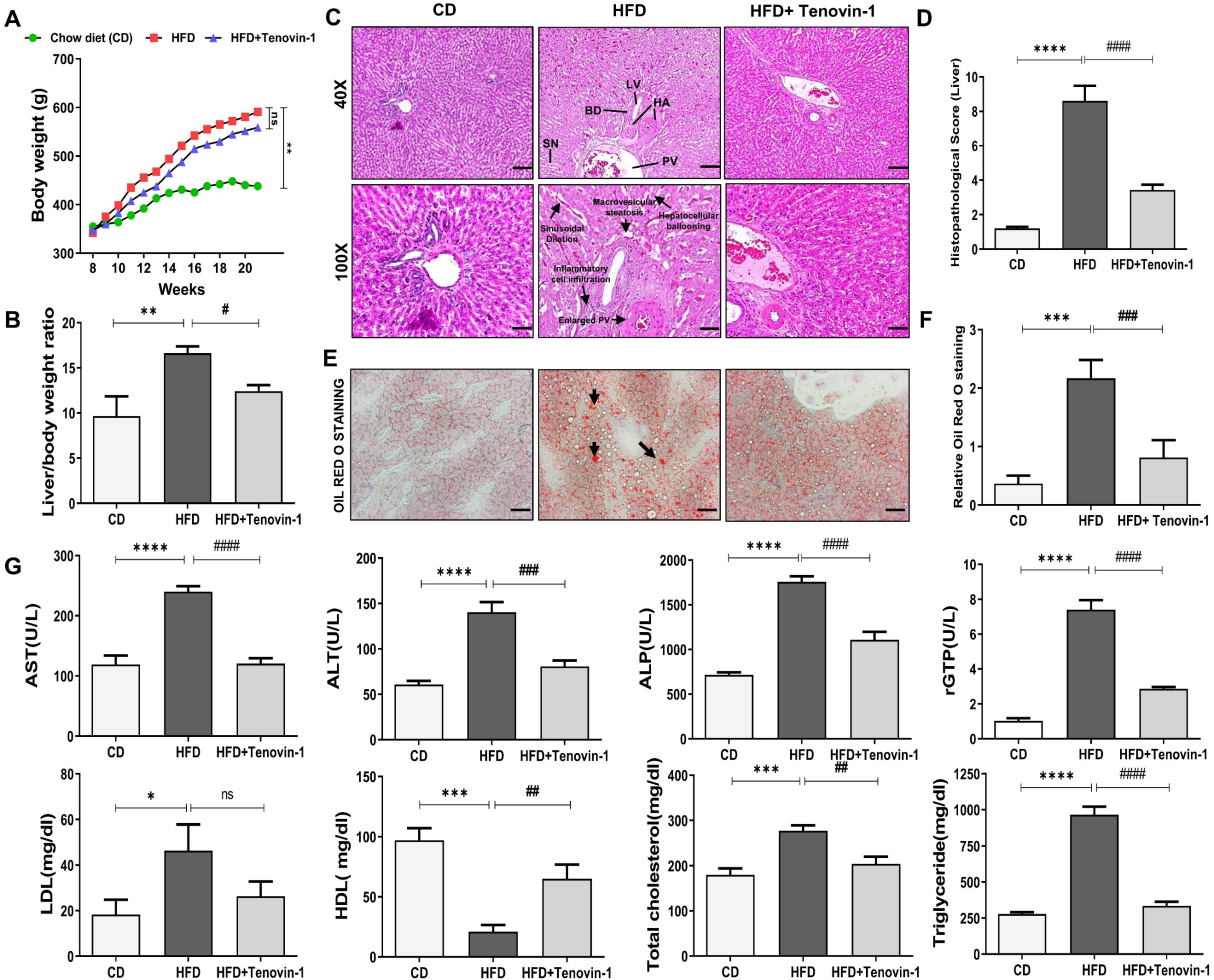
**Tenovin-1 prevents HFD-induced weight gain and hepatic injury in ZDF rats. A** Effect of Tenovin-1 on body weight changes **B** and on relative liver weights. **C** Representation of the effect of tenovin-1 on liver morphological and histological alterations in liver section by H&E. The black arrows highlight hepatic alterations owing to HFD such as expansion of portal vein, hepatocellular ballooning, sinusoidal dilation, and portal inflammation (Magnification 40X, Scale bar=200 μm; and Magnification 100X, Scale bar=100 μm). BD-Bile duct, LV-Lymphatic vessels, HA-Hepatic artery, PV-portal Vein, SN-Sinusoids **D** Histological score of liver sections after H&E staining. **E** Oil red O staining to highlight lipid storage (red) in the hepatocytes (black arrows) (Magnification 200X, Scale bar=100 μm). **F** Data quantification based on ORO staining images. **G** Measurement and representation of serum biochemical parameters: serum alanine aminotransferase (ALT), aspartate aminotransferase (AST), gamma-glutamyltransferase (rGTP), alkaline phosphatase (ALP), Triglyceride (TG), Total Cholesterol (TC), HDL (High-density lipoprotein) and low-density lipoprotein (LDL), in the liver of experimental rats. All values are expressed as the mean ± S.D. of 6 rats per experimental group (n=6). Statistical analysis was performed using one-way ANOVA followed by Bonferroni's post hoc t-test for multiple comparisons. ****p < 0.0001, ***p < 0.001, **p < 0.01, *p < 0.05 when compared with the chow diet (CD) group. ^####^p<0.0001, ^###^p<0.001, ^##^p<0.01, ^#^p<0.05, when compared to the High-fat diet (HFD) group and ns signifying non-significant difference.

The body weight of the chow diet (CD) and HFD group were monitored over a course of 20 weeks. No significant variations were observed in the body weights of rats among the three groups at baseline. Body weights of the HFD-fed group increased significantly compared to the group fed with a chow diet. The HFD group treated with Tenovin-1 had a significantly lower body weight than the HFD-fed rats after 21 weeks of HFD feeding **(Figure 2A)**. The liver weight of the rats also increased substantially in the HFD group compared with that in the CD group. Tenovin-1-treated rats showed comparatively lower liver weights than untreated rats **(Figure 2B)**. H&E staining was performed to assess hepatic steatosis, ballooning, inflammation, and fibrosis. The HFD livers showed macrovesicular and hepatocellular ballooning. Lobular and portal inflammation and sinusoidal dilation were also observed in HFD rats. Enlargement and thickening of the bile duct, portal vein, and hepatic artery were also evident in the HFD group. No significant changes were observed in the lymphatic vessels of HFD-fed rats compared to those of chow diet-fed rats. Tenovin-1-treated animals showed a substantial reduction in steatosis and hepatocyte ballooning **(Figure 2C, D)**. Morphological changes in the portal vein caused by HFD appeared to be corrected by Tenovin-1, along with a reduction in sinusoidal dilation and hepatocyte architecture. Hepatic lipid levels examined using ORO staining showed deposition of lipid droplets in HFD rats, which was, however, a lesser degree in rats treated with Tenovin-1 **(Figure 2E, F).** These data suggest the attenuation of lipid accumulation and inflammation and the restoration of the normal liver morphology by Tenovin-1.

### Tenovin-1 modulates the level of Liver enzymes in rats receiving HFD

Increased levels of liver enzymes indicate liver damage. The HFD group showed significantly higher levels of liver enzymes, including AST, ALT, ALP, rGTP, LDL, and TC and slightly lower levels of HDL than the CD group. Interestingly, the treatment with Tenovin-1 in HFD rats significantly decreased plasma AST, ALT, AL, rGTP, LDL, and TC levels and increased HDL levels. These results indicated that Tenovin-1 may have hepatoprotective potential in HFD-fed rats. HFD livers showed a significant elevation of serum TG levels compared with CD animals. However, treatment with Tenovin-1 significantly reduced TG levels in the serum, indicating the amelioration of HFD-induced lipids and lipoproteins in the liver **(Figure 2G)**.

### Tenovin-1 reduces hepatic oxidative stress, lipid peroxidation and alleviates inflammation in HFD-fed rat liver

**Figure 3:**
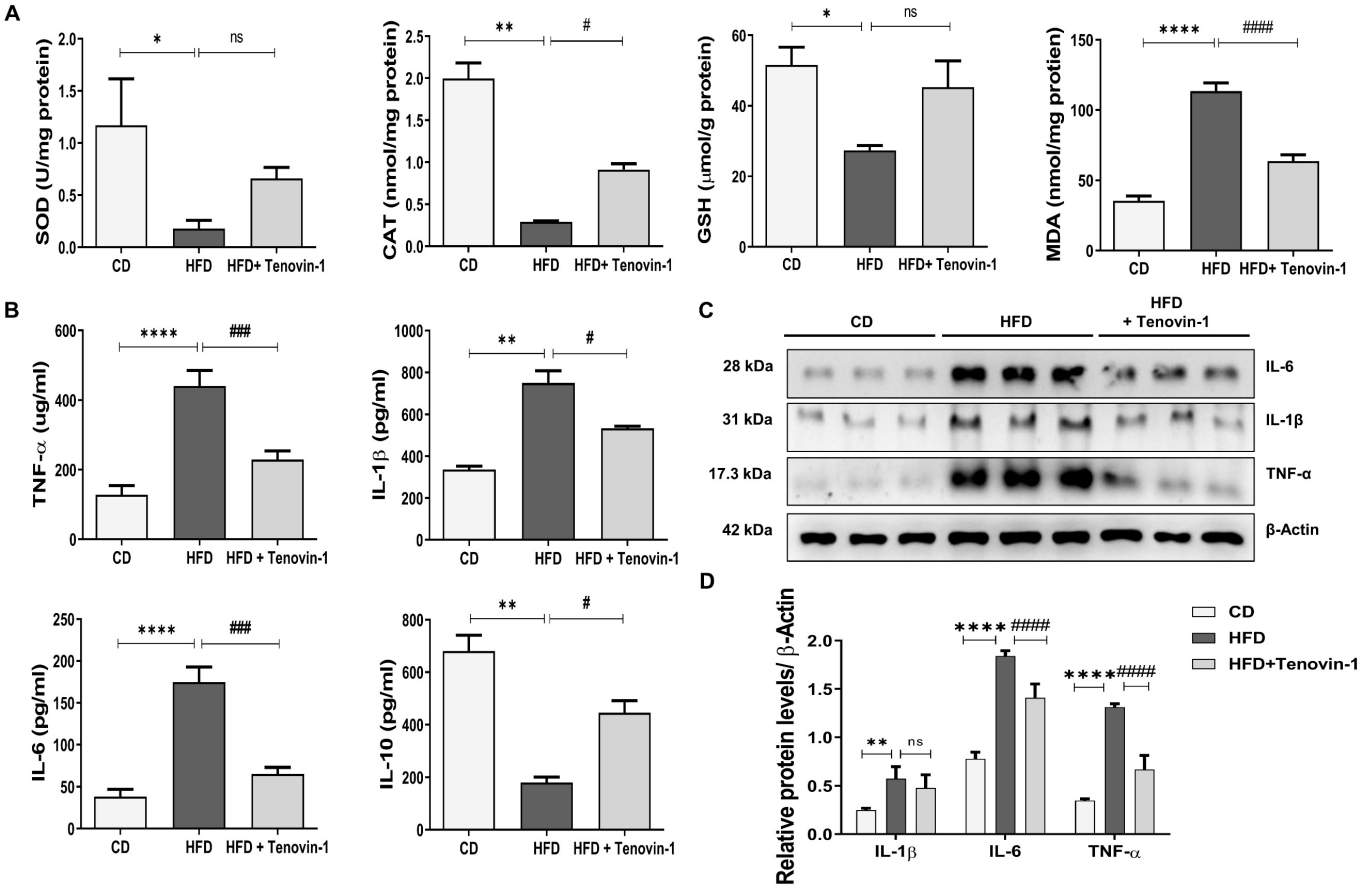
** Tenovin-1 regulates oxidative stress and inflammatory biomarkers to attenuate HFD-mediated liver fibrosis. A** The activity of oxidative stress biomarkers, superoxide dismutase (SOD), catalase (CAT), glutathione (GSH) and the content of malondialdehyde (MDA) in the liver tissues of experimental groups. **B** Representation of the levels of inflammatory cytokines, tumor necrosis factor α (TNF α), interleukin-1β (IL-1β), interleukin-6 (IL6) and interleukin-10 (IL-10) after HFD or Tenovin-1 treatment following a HFD. **C** Western blots representation and **D** quantification (ImageJ software) of the expression of inflammatory cytokines, TNF α, IL-1β and IL-10, in liver tissues of the three groups. β-Actin was used as loading control. All values are expressed as the mean ± S.D. of six rats per experimental group. Statistical analysis was performed using one-way ANOVA followed by Bonferroni's post hoc t-test for multiple comparisons. ****p < 0.0001, ***p < 0.001, **p < 0.01, *p < 0.05 when compared with the chow diet (CD) group. ^####^p<0.0001, ^###^p<0.001, ^##^p<0.01, ^#^p<0.05, when compared to the HFD group and ns signifying non-significant difference.

Sirtuins are involved in oxidative enzymatic activity 24. The activity of SOD and CAT and the concentration of GSH were measured in the presence of Tenovin-1. The activity of SOD and CAT, and the cellular level of GSH were markedly reduced in HFD-fed rats. However, these abnormalities were reversed by the administration of Tenovin-1 to HFD -fed rats. MDA levels were also measured to assess lipid peroxidation. 25. The MDA level in the HFD-fed rats was higher than that in control rats. Tenovin-1 decreased MDA levels **(Figure 3A)**.

To evaluate the effect of Tenovin-1 on inflammation, serum levels of cytokines were measured using ELISA. Tenovin-1 decreased levels of HFD-induced inflammatory cytokines such as IL-6, IL-β1 and TNF-α. In parallel, IL-10, an anti-inflammatory cytokine, decreased in the HFD liver but increased after Tenovin-1 administration **(Figure 3B)**. In addition, this inflammatory profile was confirmed by western blot **(Figure 3C, D)**.

### Tenovin-1 diminishes the expression of collagen and other ECM proteins to attenuate hepatic fibrosis in ZDF rats

**Figure 4:**
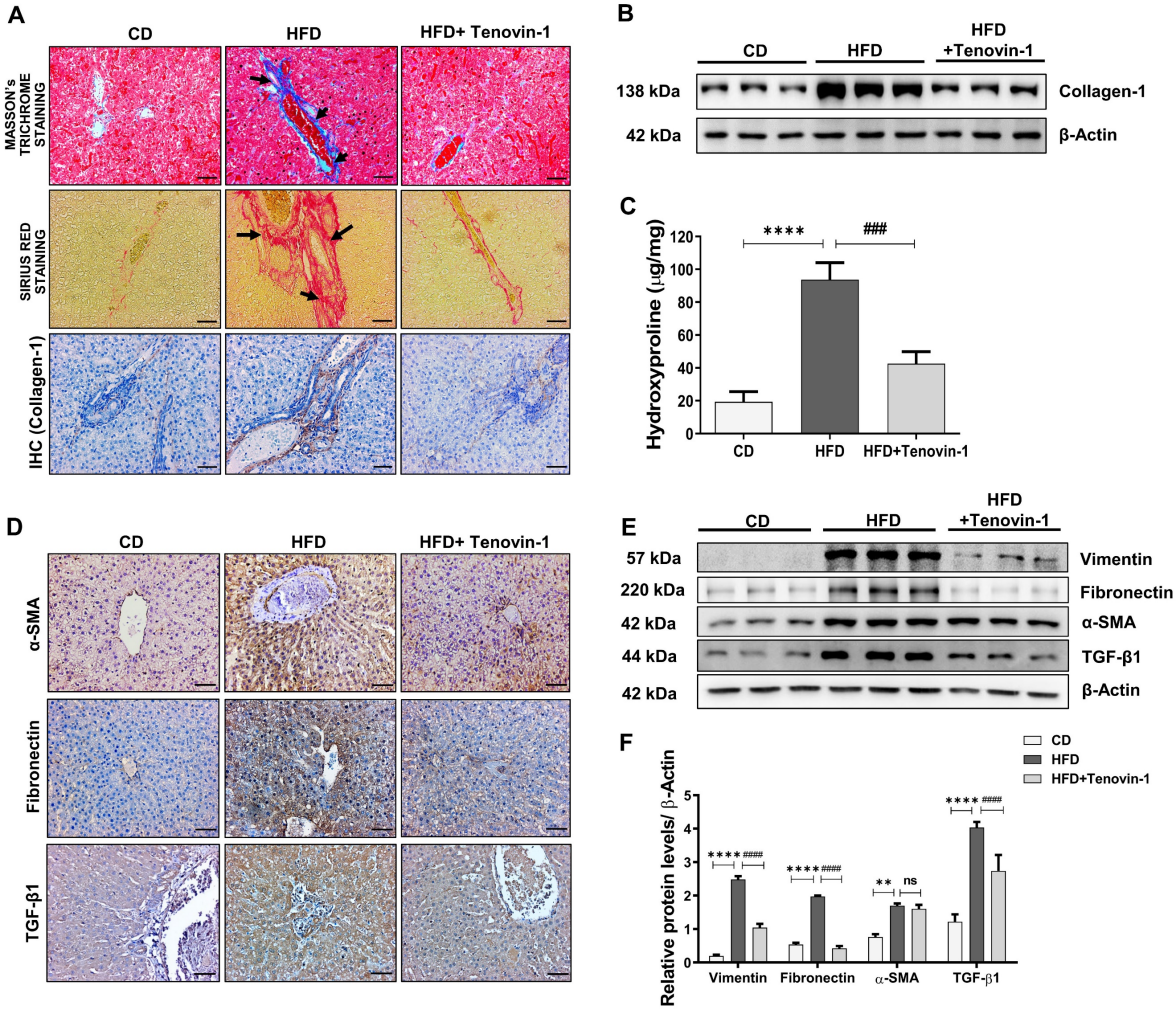
** Tenovin-1 attenuates Hepatic fibrosis by reducing deposition of collagen-1 and other fibrosis markers in the liver tissues of HFD-fed ZDF rats.** Collagen type 1 expression levels in the HFD and Tenovin-1 treated HFD group represented using **A** Masson's Trichrome staining (black arrows highlighting collagen 1 deposition), Sirius Red staining (black arrows highlighting the collagenous area), and immunohistochemical staining of collagen-1 (original magnification 200X, Scale bar=100 μm). **B** Western blot expression of collagen-1 with β-actin as loading control. **C** Representation of the concentration of 4-hydroxyproline in the livers of all experimental groups. **D** Immunohistochemical staining of α-smooth muscle actin (α-SMA), fibronectin and transforming growth factor-β1 (TGF- β1) (Original magnification 200X, Scale bar=100 μm). **E** Expression of fibrotic biomarkers, vimentin, fibronectin, α-SMA and TGF-β1 via western blot analysis using β-actin as loading control. **F** Graphical representation of the western blots using densitometric analysis and ImageJ software. All values are expressed as the mean ± S.D. of six rats per experimental group. Statistical analysis was performed using one-way ANOVA followed by Bonferroni's post hoc t-test for multiple comparisons. ****p < 0.0001, ***p < 0.001, **p < 0.01, when compared with the chow diet (CD) group. ^####^p<0.0001, ^###^p<0.001, ^##^p<0.01, when compared to the HFD group and ns signifying non-significant difference.

As pro-inflammatory cytokines are directly linked to the development of liver fibrosis 23, the reduction of IL-6, IL-β1 and TNF-α by Tenovin-1 prompted us to test its effects on hepatic fibrosis. Collagen-1 deposition is an important biomarker of liver fibrosis. Masson's trichrome (MT) staining displayed lower amount of collagen-1 accumulation in CD-fed rats whereas its deposition was significantly increased in the periportal and interstitial areas of the HFD group. However, the administration of Tenovin-1 substantially suppressed collagen-1 deposition. Sirius red staining also confirmed beneficial effects of Tenovin-1 on collagen-1 deposition in HFD-fed rats. Immunohistochemical analysis for collagen-1 was consistent with histological findings **(Figure 4A)**. The quantification in these findings is shown in **Figure S1A**. Reversal of collagen accumulation by Trenovin-1 was also confirmed through western blotting. **(Figure 4B, Figure S1B)**. Hydroxyproline is an important constituent of collagen-1 and changes in its levels often represent the onset and severity of fibrosis in the liver. An increase in hydroxyproline content was evident in HFD-fed rats, but not in Tenovin-1 treated rats **(Figure 4C)**.

Along with collagen-1, other molecular biomarkers of liver fibrosis were detected using immunohistochemical staining of liver tissue sections. The expression rate of α-SMA, TGF-β1, and Fibronectin increased in the liver tissues of HFD fed rats. However, Tenovin-1 treated rats showed comparatively lower levels of ECM proteins **(Figure 4D)**. The percentage of positively stained area is shown in **Figure S2A**. Western blot results also showed a higher expression of α-SMA, Vimentin, Fibronectin, and another important biomarker TGF-β1 fed with HFD diet. This data implied the development of fibrosis due to collagen-1 fiber and fibronectin accumulation in the liver, as marked by the presence of α-SMA and vimentin. Lower levels of these proteins were noticed in the HFD rats treated with Tenovin-1 **(Figure 4E, F)**.

### Tenovin-1 reduces apoptosis in hepatocytes of HFD-fed ZDF rats

**Figure 5:**
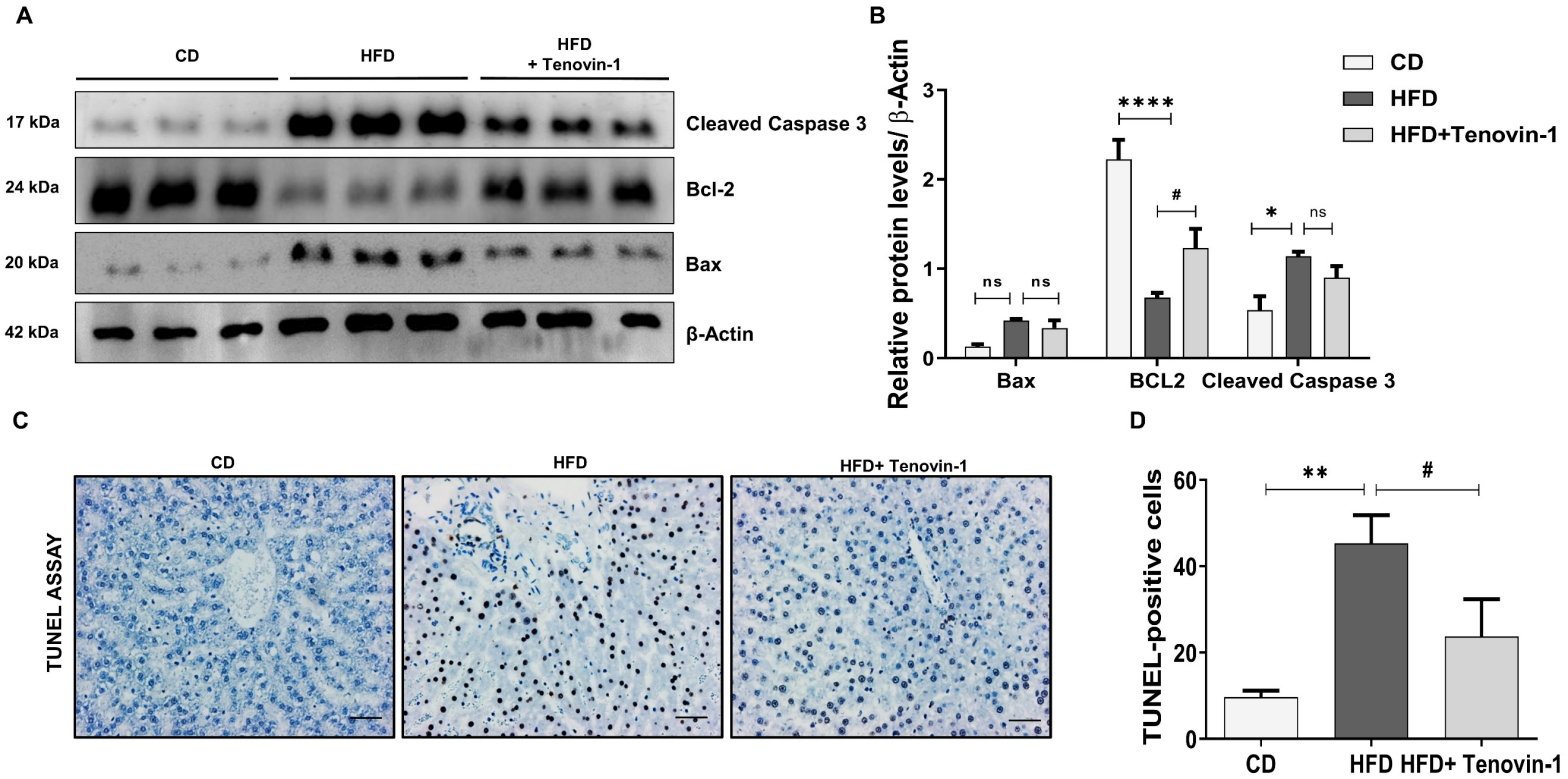
** Tenovin-1 reduces HFD-induced hepatocellular death in ZDF rats. A** Protein levels of apoptotic proteins, cleaved caspase 3, B-cell lymphoma-2 (Bcl-2), and Bcl-2-associated X (Bax) normalized against β-actin, measured using western blotting **B** Band intensities analyzed using ImageJ software, and represented as graphs. **C** TUNEL staining representing hepatocytes undergoing apoptosis (black arrows) to evaluate HFD-induced apoptosis (original magnification 200X, scale bar=100 μm). **D** Quantification of TUNEL positive cells. All values are expressed as the mean ± S.D. of six rats per experimental group. Statistical analysis was performed using one-way ANOVA followed by Bonferroni's post hoc t-test for multiple comparisons. ****p < 0.0001, *p < 0.05 when compared with the chow diet (CD) group. ^#^p<0.05, when compared to the HFD group and ns signifying the non-significant difference.

The SIRT family is involved in the regulation of apoptosis, mainly through the epigenetic regulation of histone acetylation 26. Hence, the effect of the SIRT1/2 inhibitor, Tenovin-1 on apoptotic cell death was measured. The results showed that the protein levels of Bax and cleaved Caspase 3 were higher in HFD-fed rats than in CD-fed rats. The increase of apoptotic markers declined in Tenovin-1 treated HFD rats. Bcl-2, an antiapoptotic protein, showed the opposite trend. The level of Bcl-2 was lower in the HFD group but significantly higher in the HFD+TN-1 group **(Figure 5A, B)**. The onset of apoptosis was also confirmed using the TUNEL assay, showing. The results of the TUNEL assay showed a higher number of hepatocytes undergoing apoptosis in the HFD-fed liver tissue. However, Tenovin-1 significantly reduced HFD-mediated apoptosis **(Figure 5C, D)**.

### Tenovin-1 produces anti-fibrotic effects by inhibiting TGF-β1 induced-activation of LX-2

**Figure 6:**
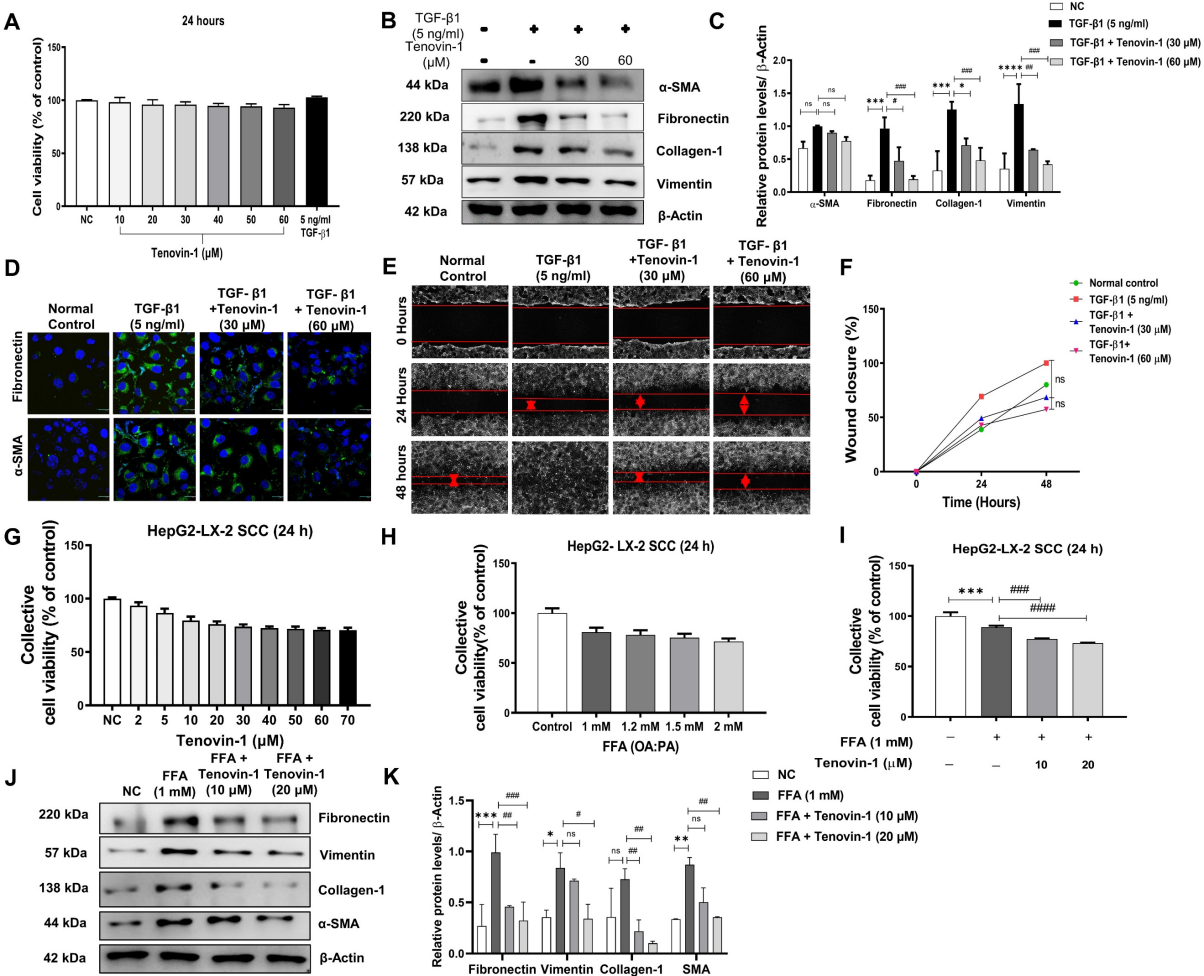
** Tenovin-1 inhibits *in vitro* hepatic stellate cell (HSC) activation.** LX-2 HSC cells were activated by TGF-β1 (5 ng/ml) and the effect of Tenovin-1 upon it was evaluated. **A** Cell viability of LX-2 cells after treatment of different concentrations of Tenovin-1 and by TGF-β1 was measured for 24 h. **B** Western blot analysis of α-SMA, fibronectin, collagen-1 and vimentin (β-actin as loading control) showing the expression of these markers upon TGF-β1-induced activation and upon Tenvoin-1 treatment at 30 μM and 60 μM **C** Densitometric analysis of western blot expressions analyzed using Image J software. **D** Immunocytochemical staining showing α-SMA, fibronectin by Tenovin-1 (30 μM and 60 μM) in TGF-β1-induced LX-2 activation. DAPI was used for nuclear staining **E** Migration of HSC cells shown by wound healing assay using Tenovin-1 (30 μM and 60 μM) in TGF-β1-induced LX-2 activation after 24 and 48 hours. **F** Graphical representation of the quantitative analysis of wound healing assay after 24 and 48 h. Simultaneous co-culture (SCC) model of HepG2 and LX-2 cells was produced, the activation of LX-2 was performed using free fatty acids (FFA) and the effect of Tenovin-1 upon it was evaluated. **G** Representation of cell viability of SCC cells after treatment of different concentrations of Tenovin-1 and by **H** FFA at different concentrations was measured for 24 h. **I** Representation of cell viability of SCC cells after treatment with FFA (1 mM) and FFA (1 mM) along with Tenovin-1 (10 μM and 20 μM). **J** Western blot expression of fibrotic biomarkers, fibronectin, vimentin, collagen-1, and α-SMA (β-actin as loading control) upon FFA-treated activation of SCC cells and their treatment with Tenvoin-1 (10 μM and 20 μM). **K** Band intensities analyzed using Image J software. All values are expressed as the mean ± S.D. of three individual experiments. Statistical analysis was performed using one-way ANOVA followed by Bonferroni's post hoc t-test for multiple comparisons. ****p < 0.0001, ***p < 0.001, when compared with the normal control group.^ ####^p<0.0001, ^###^p<0.001, ^#^p<0.05, when compared to the free fatty acid (FFA) group and ns signifying the non-significant difference.

Initially, LX-2 HSCs were treated with TGF-β1 (5 ng/ml) and different concentrations of Tenovin-1 for 24 h. No cytotoxicity was observed in LX-2 monocultured cells after the treatment with Tenovin-1 and TGF-β1 **(Figure 6A)**. Western blot analysis was performed to study the effect of Tenovin-1 on HSC activation. The expression of ECM protein markers was upregulated after the activation of LX-2 cells with TGF-β1. However, Tenovin-1 treatment downregulated this increase in a dose-dependent manner **(Figure 6B, C)**. The fluorescence of Fibronectin and α-SMA were also higher in the TGF-β1 treated LX-2 cells when compared with that of the control group. The fluorescence was reduced after treatment with Tenovin-1 **(Figure 6D)**. Activated HSCs migrate to the site of liver injury to enhance fibrogenesis. Therefore, we examined the effects of Tenovin-1 on LX-2 cell migration. TGF-β1 activated LX-2 showed a significant increase in the migration compared to that of the control group. Treatment with Tenovin-1 at 30 μM and 60 μM concentration, however, decreased the migration by TGF-β1in a dose-dependent manner **(Figure 6E, F)**.

### Tenovin-1 reduces FFA-induced fibrogenesis in a model of simultaneous cell co-culture (SCC)

LX-2 cells co-cultured with HepG2 cells were exposed to FFA and tenovin-1 at different concentrations and their cell viability was determined. Tenovin-1 shows minimal cytotoxicity in SCC cells. Concentrations of 10 μM and 20 μM were used for the experiments. A slight decrease in cell viability was observed compared to the respective control groups in FFA (OA and PA)-treated cells and cells treated with Tenovin-1 after FFA induction **(Figure 6G, H, I)**. Therefore, 1 mM concentration with minimal cytotoxicity was used in further experiments. The HepG2/LX-2 cells treated with FFA showed a significant increase in the expression of the Fibronectin, Vimentin, Collagen-1 and α-SMA, as indicated by the western blots. This implies that the activation of HSC may result from the accumulation of FFA in hepatocytes. However, Tenovin-1 reduced the expression of these proteins in a dose-dependent manner **(Figure 6J, K)**.

### Tenovin-1 regulates the expression of SIRT1/2 in HFD-fed rat liver and in FFA-treated HepG2/LX-2

**Figure 7:**
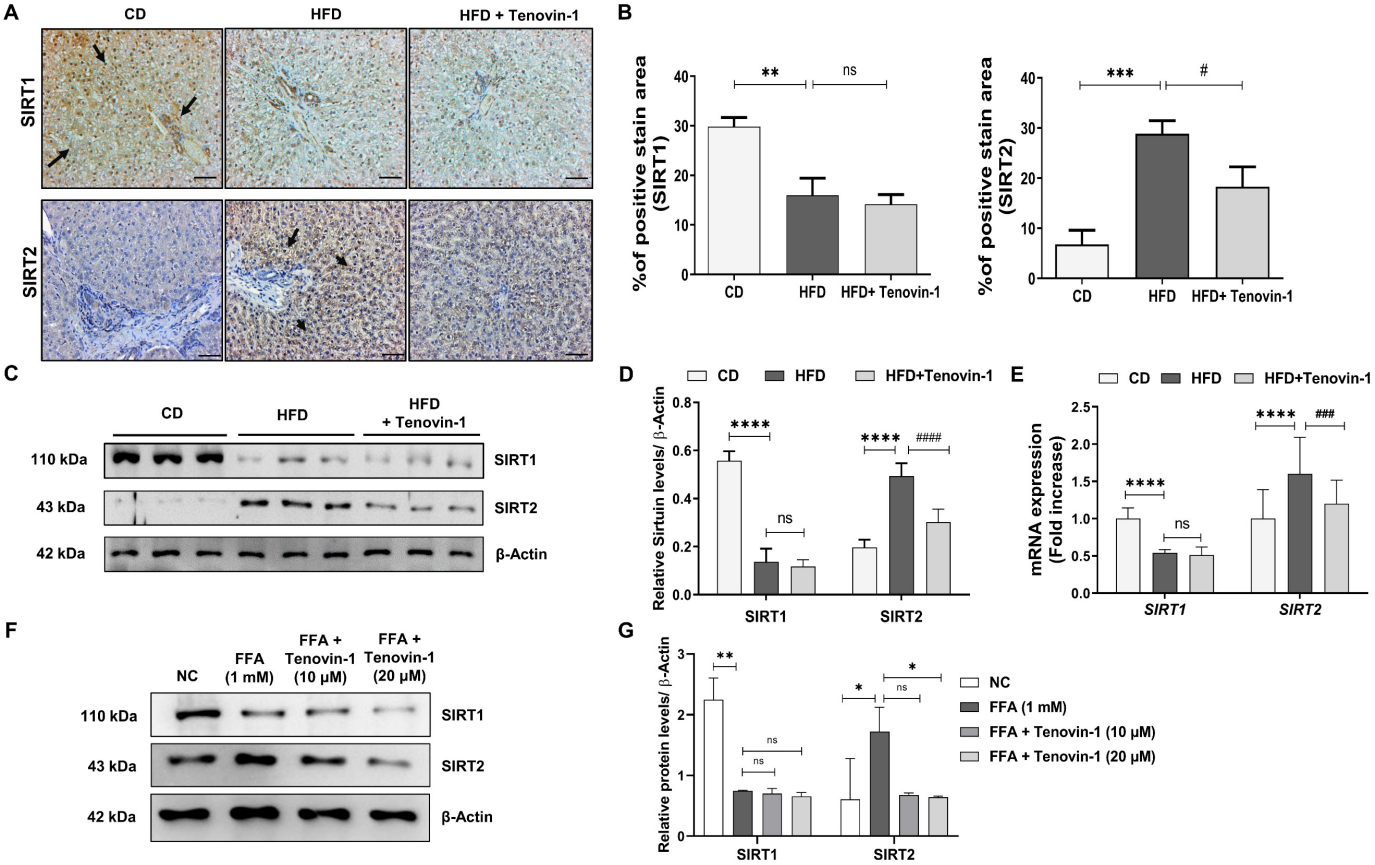
**Tenovin-1 reduces Sirtuin (SIRT) 1/2 expression in the liver of HFD rats and in FFA-treated SCC cells. A** Immunohistochemical (IHC) staining expression of SIRT1 and SIRT2 in liver tissues of ZDF rats. **B** Representation of the percentage (%) of positively stained area in IHC staining by SIRT1 and SIRT 2. **C** Expression of SIRT1 and SIRT2 via western blotting in liver tissue sections of experimental groups normalized against β-actin. **D** Relative SIRT1/2 expressions analyzed using ImageJ software. **E** mRNA levels of SIRT1 and SIRT2 genes in the liver tissue section of experimental rats. **F** Western blot analysis of SIRT1 and SIRT2 (β-Actin as loading control) in SCC cells induced by free fatty acids (FFA-1 mM) and treated by Tenovin-1 (10 μM and 20 μM). **G** Band intensities of western blot expressions analyzed using ImageJ software. All values are expressed as the mean ± S.D. of six rats per experimental group (n=6) or three individual experiments. Statistical analysis was performed using one-way ANOVA followed by Bonferroni's post hoc t-test for multiple comparisons. ****p < 0.0001, ***p < 0.001, when compared with the chow diet (CD) group or normal control group. ^####^p<0.0001, ^###^p<0.001, ^##^p<0.01, when compared to the HFD group or the free fatty acid (FFA) group and ns signifying the non-significant difference.

To understand the mechanism of action of Tenovin-1, the expression of SIRT1 and SIRT2 in the rat liver and SCC cells was analyzed. IHC revealed that SIRT1 expression was higher in the CD rats than in the HFD liver. This expression decreased non-significantly after treatment with Tenovin-1. In contrast, SIRT2 expression was higher in the HFD livers than in the CD livers **(Figure 7A, B)**. Tenovin-1-treated rats also showed a decrease in SIRT2 protein levels, confirming an inhibition of SIRT1/2 by Tenovin-1.

Western blot results were consistent with the IHC findings **(Figure 7C, D)**. Furthermore, the mRNA expression of SIRT1 was decreased while SIRT 2 was increased after HFD feeding. The expression levels of both genes decreased further upon Tenovin-1 administration, suggesting a transcriptional inhibition of *SIRT1/2* **(Figure 7E)**. Similar to the *in vivo* results, western blot analysis of the HepG2/LX-2 co-cultured cells also showed a slight decrease in SIRT1 protein, whereas an increase was observed in SIRT2 after FFA treatment. Tenovin-1, however, decreased the expression of both SIRT1 and SIRT2 in FFA-treated co-cultured cells **(Figure 7F, G)**.

### Tenovin-1 suppresses the phosphorylation of JNK and STAT3 to attenuate liver fibrosis

**Figure 8:**
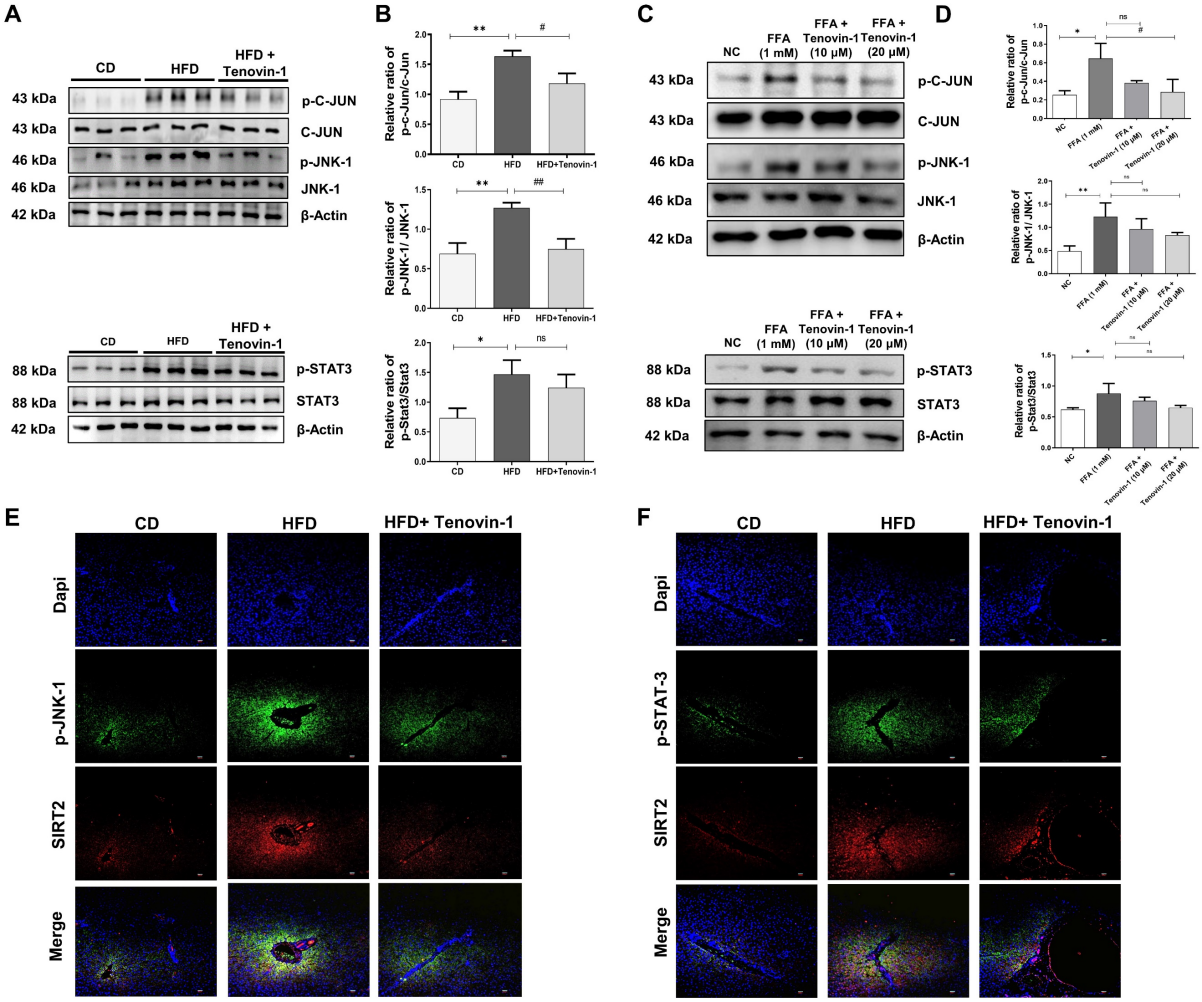
** Tenovin-1 regulates JNK-1 and STAT3 signaling to attenuate hepatic fibrosis. A** Representative western blotting analysis for expression of Jun proto-oncogene (c-JUN), p-c-JUN, c-JunNH2-terminal kinase (JNK-1), p-JNK-1, signal transducer and activator of transcription 3 (STAT3) and p-STAT3 proteins in liver section normalized against β-actin. **B** Band intensities of western blot analysis of liver sections analyzed by ImageJ software. **C** Western blot expression of c-JUN, p-c-JUN, JNK-1, p-JNK-1, STAT3 and p-STAT3 in SCC model induced by FFA and treated by Tenovin-1 (10 μM and 20 μM). **D** Relative ratio of p-c-JUN/ c-JUN, p-JNK-1/JNK-1 and p-STAT3/ STAT3 from the western blot analysis analyzed using ImageJ software. **E-F** Immunofluorescence staining of p-JNK-1 and p-STAT3 using SIRT2 for the counterstaining and DAPI for nuclear staining in the liver tissues of experimental animals. All values are expressed as the mean ± S.D. of 6 rats per experimental group (n=6) or three individual experiments. Statistical analysis was performed using one-way ANOVA followed by Bonferroni's post hoc t-test for multiple comparisons. ***p < 0.001, **p < 0.01, *p < 0.05, when compared with the chow diet (CD) or normal control group. ^##^p<0.01, when compared to the HFD group) or the free fatty acid (FFA) group and ns signifying the non-significant difference.

To study the molecular pathways involved in Tenovin-1 mediated attenuation of liver fibrosis, the expression of p-JNK was measured. JNK plays a major role in the prognosis of fibrotic disease 27. Therefore, the levels of JNK and its corresponding protein, p-c-Jun, were analyzed in the liver tissues of rats using western blotting. The results showed that p-JNK and p-c-Jun levels were higher in HFD-fed rats than in the control group. Tenovin-1 treated HFD rats showed decreased p-JNK and p-c-Jun expression. Total JNK and c-Jun levels were comparable, indicating that tenovin-1 is involved in reducing the phosphorylation of these proteins. The involvement of STAT3 in HFD-induced liver fibrosis was investigated by western blotting. STAT3 pathway regulates inflammation and apoptosis in hepatocytes 28. HFD-fed rats showed markedly higher protein expression of p-STAT3 than CD-fed rats. p-STAT3 expression decreased by Tenvoin-1. Total STAT3 expression remained unchanged in all groups **(Figure 8A, B)**.

Similar outcomes were observed in HepG2/HepG2/ LX-2 co-cultured cells treated with FFA and Tenovin-1. Western blot analysis exhibited that the expression of p-JNK and p-c-Jun increased after FFA treatment (1 mM) compared to the normal control group. However, FFA treatment followed by Tenovin-1 administration decreased the expression of both p-JNK and p-c-Jun. The expression levels of JNK and c-Jun remained unchanged after Tenovin-1 treatment. p-STAT3 expression was also increased after FFA treatment in co-cultured HepG2/LX-2 cells compared to that in normal cells. Tenovin-1 reduces p-STAT3 expression in co-cultured cells. However, the total STAT3 levels were not affected by Tenovin-1 **(Figure 8C, D)**.

Additionally, immunofluorescence was performed for p-JNK and p-STAT3, using SIRT2 as counterstaining because of its variable expression before and after treatment. The images of immunofluorescence revealed increased levels of p-JNK and p-STAT3 in the HFD group. The tenovin-1 treated HFD group showed a lower expression of JNK and p-STAT3 **(Figure 8E-F)**.

## Discussion

The coexistence of T2DM and NAFLD upsurges the risk of developing various complications such as cardiovascular disorders, chronic kidney disease, diabetic nephropathy, and diabetic retinopathy. This association is also considered bidirectional, wherein the progression of one disease increases the risk of the other 29. In patients with diabetes, the progression of NAFLD to fibrosis and cirrhosis is accelerated by obesity, inflammation, and insulin resistance 30. No effective drugs are currently available against the progression of diabetes-associated NAFLD to fibrosis. In this study, we explored how Tenovin-1 impacts HFD-induced liver fibrosis in ZDF rats.

Chronic consumption of a HFD is directly linked to the severity of NAFLD in patients with diabetes and is a key contributor to the progression of NAFLD to liver fibrosis. This occurs due to an increase in insulin resistance followed by FFA release, cell death and inflammation leading to HSC activation. Normally, rats on a HFD develop NASH-like pathology over a shorter duration of time and produce mild fibrosis-like symptoms within 24 weeks 31,32. Zucker diabetic fatty (ZDF) rats, a substrain of Zucker obese rats inbred for hyperglycemia, are diabetic, have a mutated gene encoding the leptin receptor (fa/fa), and are a well-known model for obesity-related NASH 33. Exposure of rats with pre-existing T2DM and mild steatosis to a HFD accelerates the progression of NAFLD to attain fibrosis-like characteristics 34. In our study, ZDF rats were fed a HFD with 60% fat, 20% carbohydrate, and 20% protein to exacerbate the conditions in a shorter duration and achieve an ideal model representing clinical manifestations of hepatic fibrosis. HFD caused severe disruption of cellular structures in the rat liver, accompanied with histological and morphological changes such as microvascular and macrovascular steatosis, cytoplasmic vacuolation and hepatocyte hypertrophy. Rats also exhibit hepatocellular ballooning degeneration and inflammatory cell infiltration, indicating a fibrosis-like pathology 35,36. Enlargement and hyperplasia of the liver vasculature was also observed. All combined characteristics indicated the induction of fibrosis in ZDF rats.

To study its protective effect, Tenovin-1 was given to ZDF rats at a dose of 45 mg/kg based on the study by Lian *et al.*, where the dose used in mice was converted to an animal dose using a dose calculator 19,37. Along with histopathological changes, HFD feeding in rats led to body weight and liver weight gain owing to the induction of obesity, diabetes, and NAFLD. In our study, the HFD led to a gain in body weight and hepatomegaly due to lipid accumulation in the liver of ZDF rats. In our study, the HFD led to body weight gain and hepatomegaly due to lipid accumulation in ZDF liver. Tenovin-1 in the presence of HDF reduced body weight gain. In addition, liver weights were lower than those of rats treated with tenovin-1, suggesting that tenovin-1 protects ZDF rats from hyperlipidemia and lipid accumulation, leading to a decrease in weight gain and liver weight. Leakage of liver enzymes into the bloodstream after exposure to a HFD indicates a decrease in liver function. An increase in ALT levels is related to hyperlipidemia and obesity, whereas increases in AST and ALP levels may be associated with obesity-induced diabetes. Elevated rGTP, on the other hand, is linked with steatosis and fat deposition 38. The downregulation of these enzymes after Tenovin-1 administration following HFD indicated the ablation of hepatic damage as a result of fat deposition in the liver. Moreover, FFA and cholesterol deposition also adversely affect hepatocyte function and causing lipotoxicity 39. Therefore, variations in the levels of HDL, LDL, TC, and TG in the liver are considered as biomarkers of lipotoxicity and hepatic damage. The irregular levels of these metabolic enzymes were stabilized by Tenovin-1, suggesting the normalization of liver function by tenovin-1.

Acute Intake of diet rich in fats increases β oxidation of FFAs in mitochondria increasing the flow of electrons using cytochrome-c oxidase and enhancing ROS production. Excessive ROS production by mitochondria oxidizes unsaturated lipids in fat deposits and causes lipid peroxidation, with MDA as the final product. This leads to the release of reactive aldehydes, which increases hepatic fibrogenesis 40. Insulin resistance or diabetic state along with metabolites such as FFAs and specific cytokines can also induce ROS production and subsequent inflammation. However, antioxidants such as SOD, CAT, and GSH can remove ROS and lipid peroxidases and protect the liver from oxidative stress 41. In the present study, the reduction in SOD, CAT, and GSH levels suggested that oxidative stress was due to excessive lipid deposition. In contrast, an increase in MDA levels also hinted at an increase in lipid peroxidation after the administration of HFD. The restoration of antioxidant enzymes and amelioration of MDA levels after tenovin-1 administration can be attributed to its antioxidant properties. Therefore, it can be considered that Tenovin-1 might have beneficial effects against oxidative stress. Furthermore, HFD-mediated oxidative stress is responsible for triggering inflammation through the production of inflammatory cytokines. Increased caloric intake amasses visceral adipose tissue (VAT) which is responsible for producing proinflammatory cytokines and is linked to a reduction in anti-inflammatory adiponectin 42. HFD-induced IKK-β activation triggers NF-κB activation and results in a subsequent increase in inflammatory mediator production including TNFα, IL-6, and IL-1β 43. IL-10, on the other hand is considered as a negative regulator in inflammatory process and is responsible for the inhibition of secretion of inflammatory mediators such as TNFα, IL-6 by the hepatic macrophages 44. Kupffer cells, resident liver macrophages, are activated by a HFD or FFA treatment to produce pro-inflammatory cytokines and contribute to fibrogenesis 45. In our study, an increase in TNFα, IL-6, and IL-1β and a decrease in IL-10 upon HFD administration was observed. Tenovin-1, however, reduced the levels of pro-inflammatory cytokines and increased IL-10 levels, suggesting an anti-inflammatory effect of tenovin-1 in HFD-fed rats.

Chronic injury to liver cells and prevailing fibrogenic response by HSCs are the major causes of liver fibrosis. Activation of these HSCs results in the production of type 1 collagen, causing it to deposit in the ECM within the sinusoids 46. Therefore, collagen 1 is considered a marker of liver fibrosis. Previous studies confirmed an increase in collagen-1 deposition after administration of a HFD 47. Similarly, in our study, collagen-1 deposition was observed in the livers of rats fed with HFD. However, Tenovin-1 treated rats showed lesser collagen 1 than the untreated rats. In addition, among the chemokines and various growth factors secreted during HFD-induced inflammation, TGF-β1 is the key mediator of fibrogenesis, as it is involved in the proliferation and activation of HSCs. Apoptotic hepatocytes release TGF-β1 during liver damage to activate quiescent HSCs and potentiate their trans-differentiation into myofibroblasts 48. Thus, TGF-β1 signals for the abundant synthesis and secretion of ECM proteins such as collagen 1 and fibronectin. Upregulated expressions of mesenchymal markers such as α-SMA and vimentin (intermediate filament protein) are also considered as reliable biomarkers indicating activation of HSCs followed by ECM deposition 49. Our study showed an increase in the expression of TGF-β1 along with an upregulation in the expression of both pericellular matrix protein markers (α-SMA and vimentin) and other ECM protein marker (fibronectin). Tenovin-1 significantly reduced the expression of TGF-β1 and subsequent fibrotic proteins, implying a reduction in the degree of fibrosis in the liver mediated via TGF-β1 reduction. We also used LX-2 cells derived from an activated immortalized hepatic stellate cell line that exhibited characteristics of activated HSCs. They were treated with TGF-β1 to enhance or sustain their activation and model fibrotic responses. TGF-β1 treatment increased proliferation, migration, and fibrogenesis. A dose-dependent reduction in the proliferation and migration of these cells was observed upon Tenovin-1 treatment, indicating a reduction in their migration to the injury site, thus delaying fibrosis. However, a decrease in the expression of fibrotic proteins indicated the anti-fibrotic effect of Tenovin-1. It has been shown that chronic accumulation of lipid droplets in hepatocytes activates fibrogenic HSC via cytokine production 50. A simultaneous co-culture (SCC) of hepatocytes with HSCs mimics early fibrogenesis following free fatty acid (FFA) overload 51,52. FFA treatment also upregulates the expression of fibrosis-related proteins. In our study, Tenovin-1 reduced the expression of these fibrotic proteins in the SCC cell line, similar to the *in vivo* findings. Tenovin-1 can therefore, attenuate the activation of HSC under the condition of lipotoxicity. The correlation between the *in vitro* and *in vivo* results suggest that Tenovin-1 might exhibit an anti-fibrotic effect by inhibiting HSC activation.

Hepatocellular apoptosis is attributed to HFD-mediated oxidative stress. The accumulation of fatty acids and free cholesterol after HFD feeding results in various cellular processes such as mitochondrial dysfunction, and insulin resistance resulting in hepatocyte apoptosis. The subsequent initiation of apoptotic pathway upregulates pro-apoptotic proteins such as Bax, pro-caspases (cleaved caspase 3) and downregulates anti-apoptotic proteins such as Bcl-2, leading to apoptosis 53. Our study showed that hepatocytes underwent apoptosis upon a HFD consumption in a similar manner. This is due to the accumulation of fats, leading to excessive production of ROS in the mitochondria and causing mitochondrial dysfunction, which is an important sign of apoptosis and is controlled by Bcl-2 family proteins. The release of these apoptotic proteins such as Cyt *c* from the mitochondria activates downstream effector Caspase-3, causing apoptosis. Therefore, the anti-apoptotic effect of Tenovin-1 can probably be ascribed to the inhibition of oxidative stress mediated by Tenovin-1.

The role of SIRT1 and SIRT2 in NAFLD and liver injury is certain and continues to be a growing area of research. Generally, SIRT1 suppression has been associated to liver injury and its upregulation can protect against Hepatic damage. Numerous SIRT1 activators have been used studied for their protective role in the liver. For instance, a SIRT1 activator resveratrol showed protective effects in the livers of mice fed ethanol by activating the SIRT1-AMPK signaling pathway 54. SIRT2, on the other hand, can aggravate liver injury and its inhibition has been found to decrease hepatic fibrosis 16. A study also showed that the deficiency of *Sirt2* alleviated hepatic fibrosis in thioacetamide (TAA) and carbon tetrachloride (CCl_4_) induced Hepatic fibrosis 13. Zhou *et al.* 55 stated in their study that AK-1, (SIRT2 inhibitor) alleviated hepatotoxicity induced by carbon tetrachloride. Overall, this effect of SIRT1 and SIRT2 in liver diseases including NAFLD, has been observed to be similar. The effect of several dual SIRT1/2 inhibitors have also shown positive outcomes in liver diseases. Studies show the protective effect of Suramin, a potential SIRT1/2 inhibitor, against liver damage 56. Another inhibitor of SIRT1 and SIRT2, Sirtinol, attenuated hepatic injury induced by trauma hemorrhage in rat model 57. Despite their protective action in the liver, an explanation of SIRT1/2 inactivation leading to protective effect in liver has not been provided in these studies. In an attempt of a better understanding of SIRT1/2 and their inhibitors, we evaluated the effect of Tenovin-1, on HFD-induced Hepatic Fibrosis in ZDF rats and an FFA-treated SCC model. Tenovin-1 is a potent inhibitor of SIRT1/2 deacetylating activity and an increase in SIRTs could not be a possible outcome as Tenovin-1's response to hepatic injury, the protective action of Tenovin-1 against hepatic fibrosis can be attributed to its reduction in SIRT1/2 activity. An overall, suppression of SIRT1 and SIRT2 expression was observed after Tenovin-1 treatment in both models. Interestingly, Tenovin-1 administration resulted in a non-significant reduction in SIRT1 levels and in contrast, higher SIRT2 expression in HFD-induced fibrotic rats was significantly reduced by Tenovin-1 13. Tenovins show a lower potency against SIRT1 and has a slightly higher selectivity towards SIRT2 58. Previously conducted research suggests that although Tenovin-1 causes inhibition of both SIRT1 and SIRT2, SIRT2 inhibition is more potent in its activity. The study evaluating its effectiveness in mammalian cells, indicated that SIRT2 was more prominently inhibited by Tenovin-1. It is possible that SIRT2 plays a major role in its cellular and biological activity. Since studies have shown that SIRT2 inactivation can protect the liver from hepatic injury, in the current study, the protective action of Tenovin-1 against hepatic fibrosis can be attributed to its potent reduction in SIRT2 activity rather than a non-significant reduction in SIRT1 16, 59. Therefore, a potent SIRT2 inhibition over a mild SIRT1 inhibition by Tenovin-1 could also be responsible for the positive hepatoprotective effects of tenovin-1 in response to a high fat diet. Additionally, considering that both SIRT1 and SIRT2 are NAD+ (Nicotinamide adenine dinucleotide) dependent protein deacetylases (HDACs), acting on both histones and non-histones by catalyzing the removal of acetyl functional group from lysine residue, Tenovin-1 acts by inhibiting this dual deacetylase activity of SIRT1/2 5. Since inhibition of HDACs has been known to reverse myofibroblast differentiation during liver fibrosis, similar inhibition of deacetylation activity by Tenovin-1 might be involved in the reduction of Hepatic fibrosis in the present study. According to our hypothesis, it is possible that using SIRT1/2 inhibitors reduce the deacetylation activity of HDACs, causing acetylation and leading to a reduction in the phosphorylation of certain signaling proteins to reduce fibrosis 60, 61. The SIRT1 downregulation having a protective effect on the liver might be a result of its combined phosphorylation inhibition of the involved signaling proteins along with a positive contribution of SIRT2 inhibition. However, the explanation to how other SIRT1/2 inactivators produce their effects remains a question for further studies.

SIRT1/2 may also target intracellular signaling pathways to regulate liver fibrosis. Sarikhani *et al.* 62 found that SIRT2 deacetylates c-JunNH2-terminal kinase (JNK). Deacetylation by SIRT2 enhances the enzymatic activity of JNK towards c-JUN and favors JNK phosphorylation. It also promotes ROS-induced cell death. Another related study increased apoptosis to JNK activation in a HFD-induced NAFLD model 63. Since our study showed the upregulation of c-JUN and JNK phosphorylation in both HFD- and FFA-treated SCC models, their upregulation might be an indicator of apoptosis induced by oxidative stress, as shown previously. Conversely, a reduction in their expression upon tenovin-1 treatment is an indication of the attenuation of apoptosis in hepatocytes, and ultimately a reduction in liver fibrosis. Various studies have shown effects similar to those of SIRT2, in which SIRT1 reduction blocked oxidative stress-mediated apoptosis by downregulating JNK 64. However, a contributory relationship between SIRT1 and JNK still remains to be elucidated.

SIRTs, being HDAC, may be involved in the phosphorylation of STAT3 and STAT3-mediated increase in ECM production during fibrosis 65. Several HDAC inhibitors have been known to inhibit STAT3 signaling to mediate antifibrotic effects 66, 67. Our results also showed an increase in STAT3 phosphorylation upon HFD administration, similar to the study conducted by C. Fang *et al.* 68. Tenovin-1 reduced this STAT3 phosphorylation increased by HFD administration. As discussed before, the inhibition of deacetylase activity and further acetylation causes reduction in phosphorylation. It can be speculated that the reduction in STAT3 phosphorylation by tenovin-1 could result from its deacetylase inhibitory activity which further leads to a decrease in the expression of fibrosis-associated genes. Given that STAT3 can also be either pro- or anti-inflammatory in response to HFD, the production of inflammatory signals depends on the progression of injury 28. In our study, we observed that STAT3 played a pro-inflammatory role, which leads to the pathogenesis of liver fibrosis, and is inhibited by Tenovin-1. Therefore, the anti-inflammatory role of Tenovin-1 may be attributed to the inhibition of STAT3 phosphorylation.

Despite the advancements and multiple clinical trials, no potential treatments other than stearyl-CoA desaturase (SCD)1 inhibitors have shown positive results in hepatic fibrosis patients with T2DM 69. We studied a SIRT1/2 inhibitor, Tenovin-1, as a novel intervention for the treatment of hepatic fibrosis in diabetic rats. The results showed Tenovin-1 is an effective option against hepatic fibrosis and the factors which lead to its progression such as oxidative stress and inflammation. The given dose of Tenovin-1 was well-tolerated by the rats highlighting its safety and tolerability. Elaborately, Tenovin-1 showed a protection against High fat diet-induced weight gain, steatosis, oxidative stress, inflammation, hepatocyte apoptosis and fibrosis in ZDF rats causing a reduction in progression of NAFLD to fibrosis. An inhibition of HSC activation induced by TGF-β1 and free fatty acid accumulation in hepatocytes showed its ability for HSC inactivation. Tenovin-1 targets the phosphorylation JNK and STAT3 to inhibit oxidative stress mediated-hepatic apoptosis and inflammation. The inflammatory mediators such as TGF-β1 activate HSCs from their quiescent state to form myofibroblasts leading to excessive ECM accumulation and fibrosis in the liver. Therefore, targeting JNK and STAT3 by Tenovin-1 inhibits this activation of HSCs, hence, decreases hepatic fibrosis. Our findings suggests that Tenovin-1 might serve as a viable treatment option for hepatic fibrosis and a potential agent to be used clinically for better patient outcomes. Although, our study was able to relate the effect of Tenovin-1 to its individual SIRT1 and SIRT2 deacetylase activity, a proper validation of simultaneous *SIRT1/2* inhibition by double gene knockdown study can provide further insight of their effect on liver function and fibrosis, which was limited in our study. This may result in the assessment and comparison of other dual SIRT1/2 inhibitors in diverse fibrotic conditions. Additionally, as the treatment of liver fibrosis can continue over a long course of time, trials are required to assess the safety of Tenovin-1, along with its efficacy, in those patients with hepatic fibrosis with or without metabolic conditions. Therefore, further studies are required to be conducted to offer novel insights regarding the use of Tenovin-1 as an anti-fibrotic medication.

## Supplementary Material

Supplementary figures and table.

## Abbreviations

ALT: alanine aminotransferase
ALP: alkaline phosphatase
AST: aspartate aminotransferase
ATCC: American type culture collection
BCA: bicinchoninic acid
BCL 2: B-cell lymphoma protein 2
BSA: bovine serum albumin
Bax: BCL2 associated X
CAT: catalase
CD: chow diet
CO_2_: carbon dioxide
DAB: 3,3'-diaminobenzidine
DAMPs: damage-associated molecular patterns
DAPI: 4′,6-diamidino-2-phenylindole
DMEM: Dulbecco's minimum essential medium
DMSO: dimethyl sulfoxide
DW: deionized water
ECM: extracellular matrix
EDTA: ethylenediaminetetraacetic acid
ELISA: enzyme-linked immunosorbent assay
EMT: epithelial-mesenchymal transition
EndMT: endothelial to mesenchymal transition
FBS: fetal bovine serum
FDA: food and drug administration
FFA: free fatty acid
FMT: fibroblast-to-myofibroblast transition
GSH: glutathione
rGTP: gamma-glutamyltransferase
H4K16: H4 histone at lysine 16
H&E: Haematoxylin and eosin
HCC: hepatocellular carcinoma
HDAC: histone deacetylases
HDL: high density lipoprotein
HFD: high-fat diet
HSC: hepatic stellate cells
HRP: horseradish peroxidase
GSH-Px: glutathione peroxidase
IHC: immunohistochemical
IKK-β: IkappaB kinase
IL-1β: interleukin-1β
IL6: interleukin-6
IL-10: interleukin-10
JNK-1: c-JunNH2-terminal kinase
c-JUN: Jun proto-oncogene
LDL: low density lipoprotein
LX-2: Lieming Xu-2
MDA: malondialdehyde
mRNA: messenger ribonucleic acid
MT: Masson's trichrome
mTOR: mechanistic target of rapamycin
NAD: nicotinamide adenine dinucleotide
NAFLD: non-alcoholic fatty liver disease
NASH: non-alcoholic steatohepatitis
NF-κB: nuclear factor kappa-light-chain-enhancer of activated B cells
ns: non-significant
OA: oleic acid
PA: palmitic acid
PAMPs: pathogen- associated molecular patterns
PBS: phosphate buffer saline
PBST: phosphate-buffered saline with 0.1% Tween 20 Detergent
PVDF: polyvinylidene fluoride or polyvinylidene difluoride
RIPA: radioimmunoprecipitation
RNA: ribonucleic acid
RT-PCR: reverse transcription polymerase chain reaction
SCC: simultaneous co-culture
SD: standard deviation
SDS-PAGE: sodium dodecyl sulfate-polyacrylamide gel electrophoresis
SIRT: sirtuin
SOD: superoxide dismutase
ROS: reactive oxygen species
STAT3: signal transducer and activator of transcription 3
α-SMA: α-smooth muscle actin
T2DM: Type 2 diabetes mellitus
TBARS: thiobarbituric acid reactive substances
TBS: tris-buffered saline
TC: total cholesterol
TdT: Terminal Deoxynucleotidyl Transferase
TG: triglyceride
TGF-β1: transforming growth factor-β1
TMB: 3,3′5,5′-tetramethylbenzidine
TNF α: tumor necrosis factor α
TN-1: Tenovin-1
TUNEL: apoptotic terminal deoxynucleotidyl transferase dUTP nick end labeling
UV: ultra-violet
WB: western blot
Wnt: wingless/integrated
WST-1: water-soluble tetrazolium salt
ZDF: zucker diabetic fatty
