# Supplementary material for: Tenovin-1, a Selective SIRT1/2 Inhibitor, Attenuates High-fat Diet-induced Hepatic Fibrosis via Inhibition of HSC Activation in ZDF Rats
Source: Int J Biol Sci. 2024 Jun 11;20(9):3334–52. doi: 10.7150/ijbs.97304 (PMC11234213; doi:10.7150/ijbs.97304)
Supplement: Supplementary file 1 — Supplementary figures and table. [file ijbsv20p3334s1.pdf]

**SIRT1**	**Forward**	**GAA CCT CTG CCT CAT CTA**
	Reverse	GCA TAC TCG CCA CCT AAC
**SIRT2**	Forward	TGG TGG GTC CTA TTG TCG
	Reverse	GTG TCC GAG TCT GAA TCCT
**β-actin**	Forward	CGT TGA CAT CCG TAA AGA CC
	Reverse	TAG AGC CAC CAA TCC ACA C

Table S1: Primer sequences (Sense, Anti-sense: 5′ to 3′) of genes used for RT-PCR analysis

A

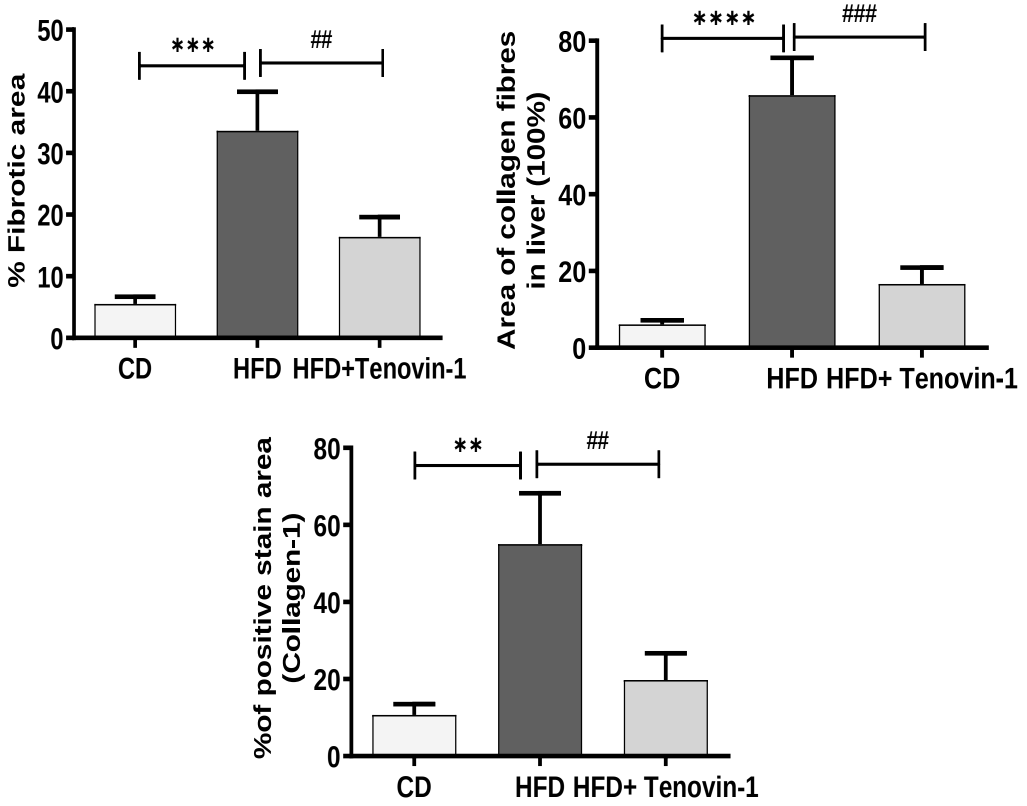

B

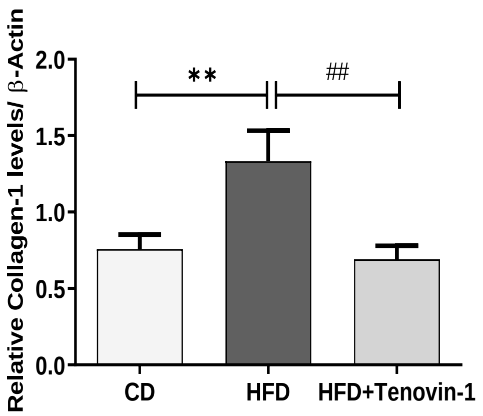

Figure S1 A: Graphical representation of the percentage (%) area of Collagen-1 fibers stained using Masson's Trichrome staining in the liver sections, percentage (%) fibrotic area of collagen-1 stained by Sirius Red staining in the liver sections and percentage positive area of collagen- 1 stained using collagen-1 antibody in immunohistochemical staining of the liver sections. **B** Band intensity of western blot expression of collagen-1 analyzed using ImageJ software. All values are expressed as the mean ± S.D. of six rats per experimental group (n=6). Statistical analysis was performed using one-way ANOVA followed by Bonferroni's post hoc t-test for multiple comparisons. ****p < 0.0001, ***p < 0.001, **p < 0.01, when compared with the chow diet (CD) group. $^{###}$p<0.001, $^{##}$p<0.01, when compared to the High fat diet (HFD) group).

A

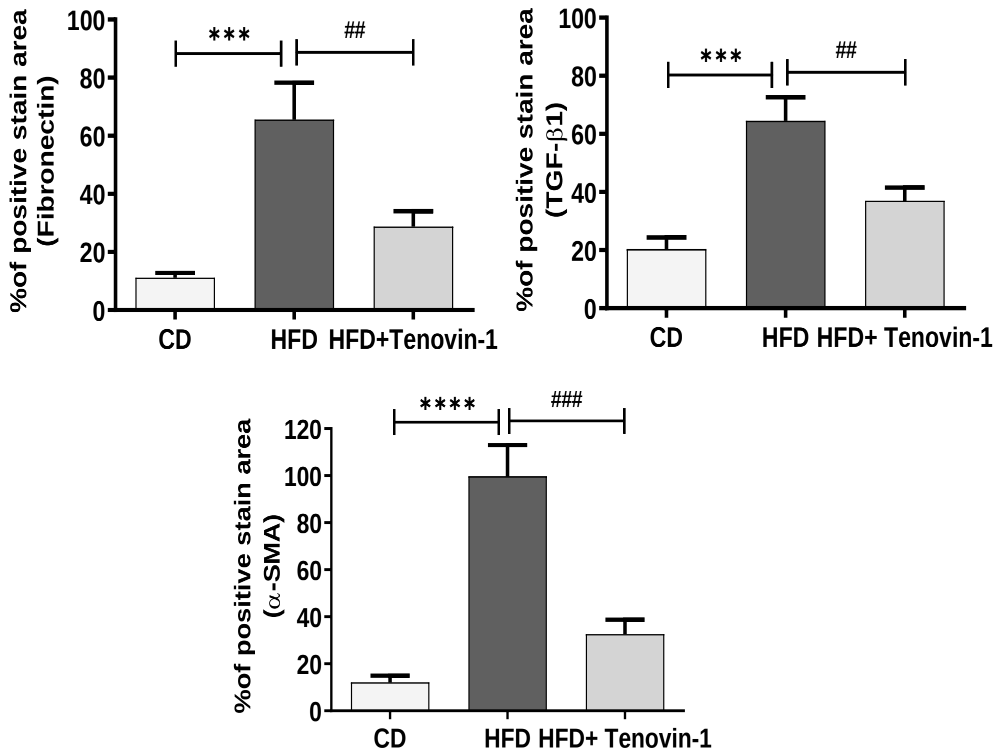

Figure S2 A: Graphical representation of percentage (%) positive area stained using α-SMA, fibronectin and TGF-β1 antibody in immunohistochemical (IHC) staining of the liver sections of all experimental groups. All values are expressed as the mean ± S.D. of six rats per experimental group (n=6). Statistical analysis was performed using one-way ANOVA followed by Bonferroni's post hoc t-test for multiple comparisons. ****$p < 0.0001$, ***$p < 0.001$, when compared with the chow diet (CD) group. ###$p<0.001$, ##$p<0.01$, when compared to the High fat diet (HFD) group).

A

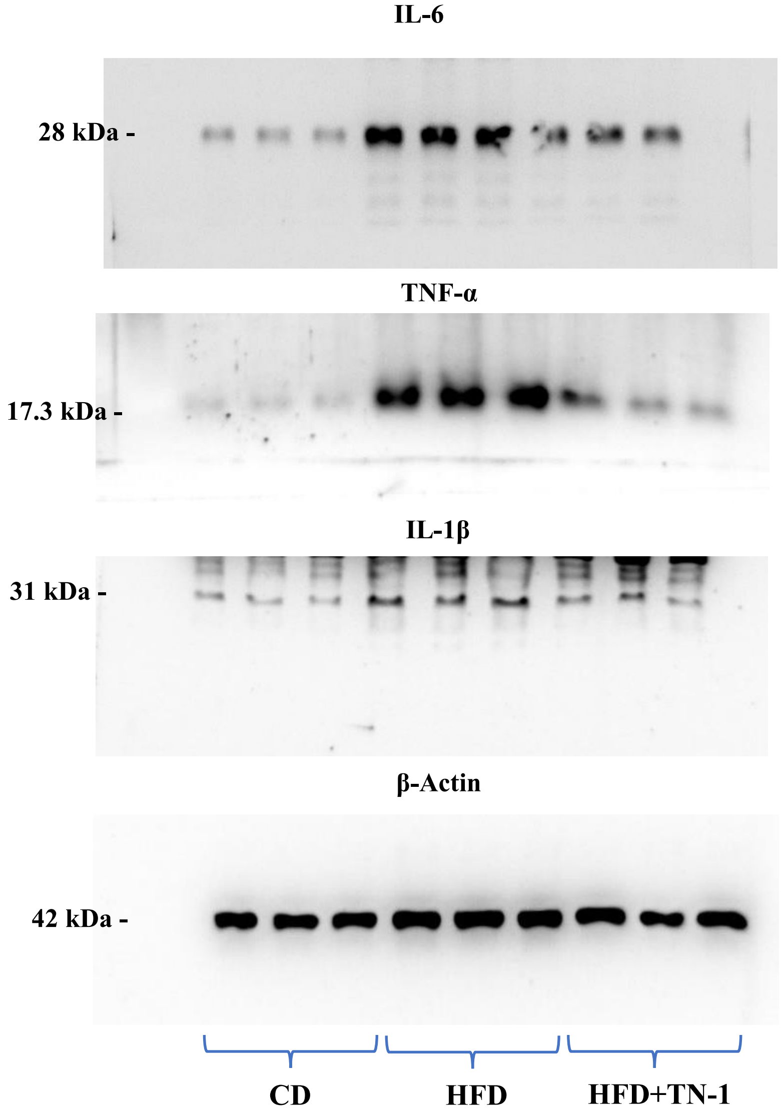

Figure S3 A: Whole blot images of IL-6 (28 kDa), IL-1β (31 kDa), TNF-α (17.3 kDa) and β-Actin (42 kDa) as represented in Figure 3C.

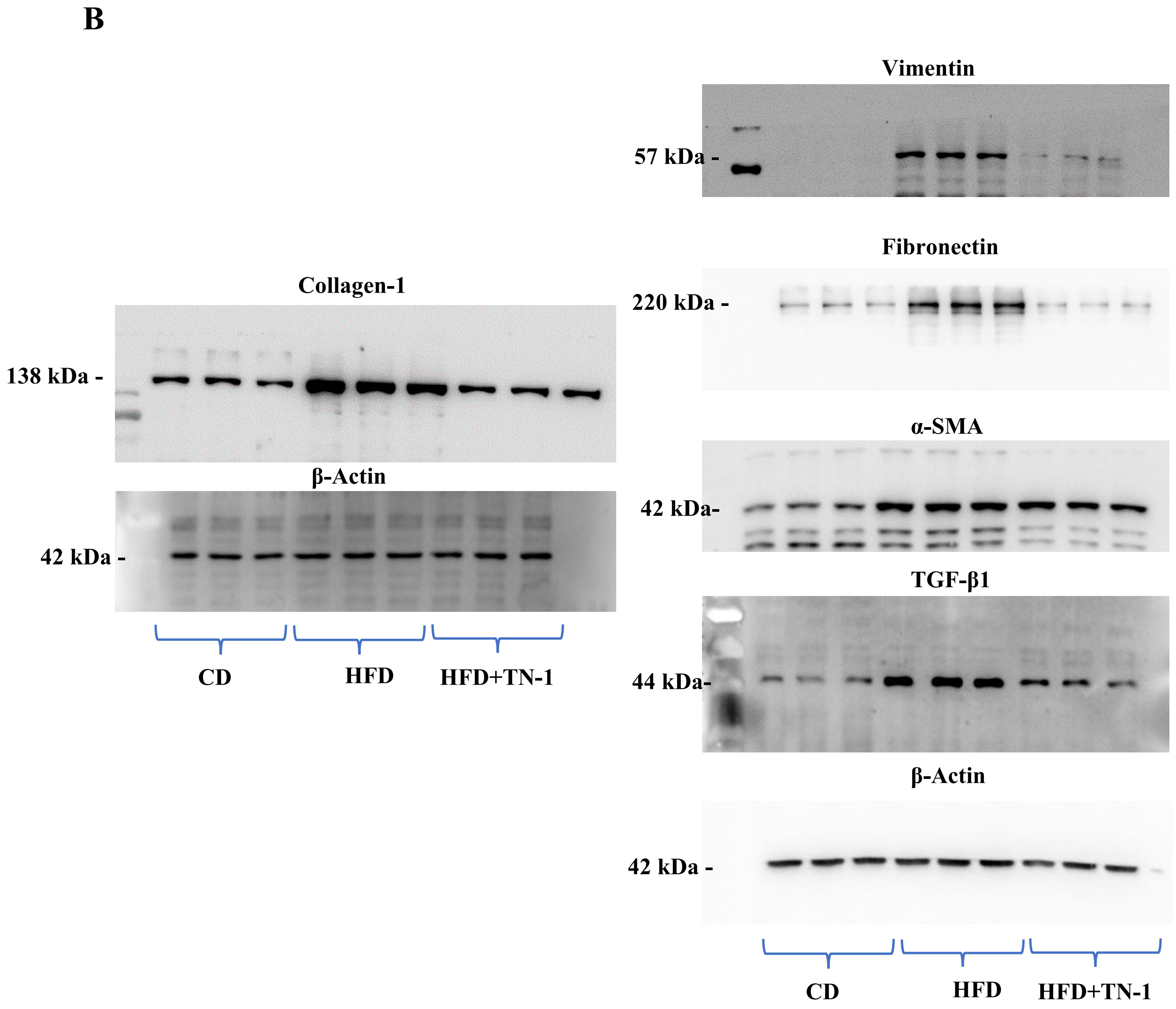

Figure S3 B: Whole blot images of Collagen-1 (138 kDa), Vimentin (57 kDa), Fibronectin (220 kDa), α-SMA (42 kDa), TGF-β1 (44kDa) and β-Actin (42 kDa) as represented in Figure 4B, E.

C

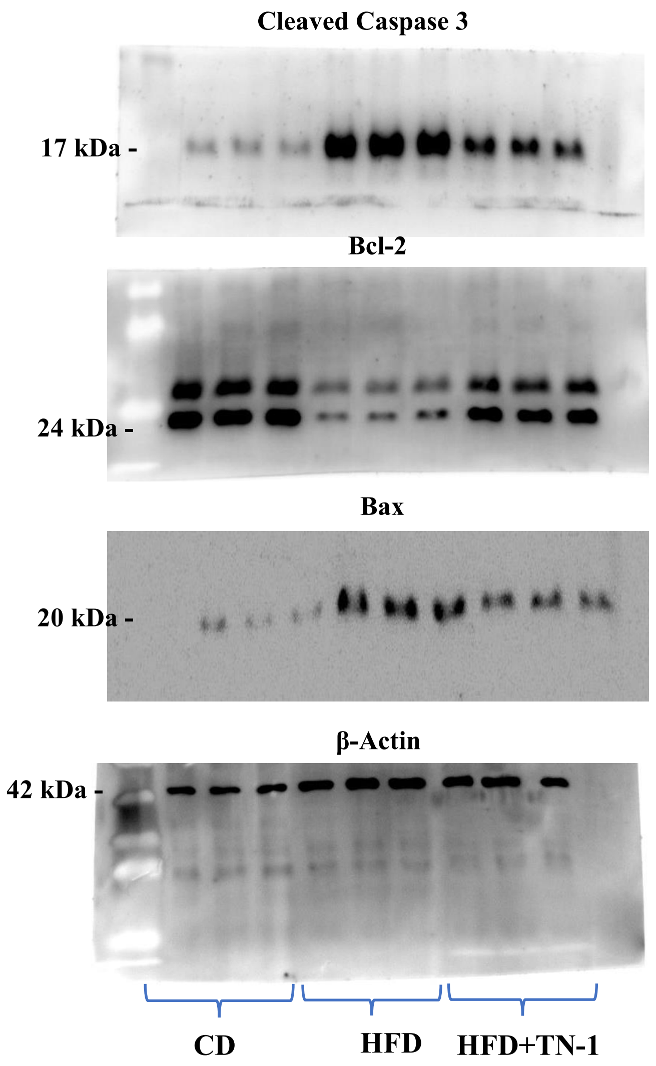

Figure S3 C: Whole blot images of **Cleaved Caspase 3** (17 kDa), **Bcl-2** (24 kDa), **Bax** (20 kDa) and β-Actin (42 kDa) as represented in Figure 5A.

D

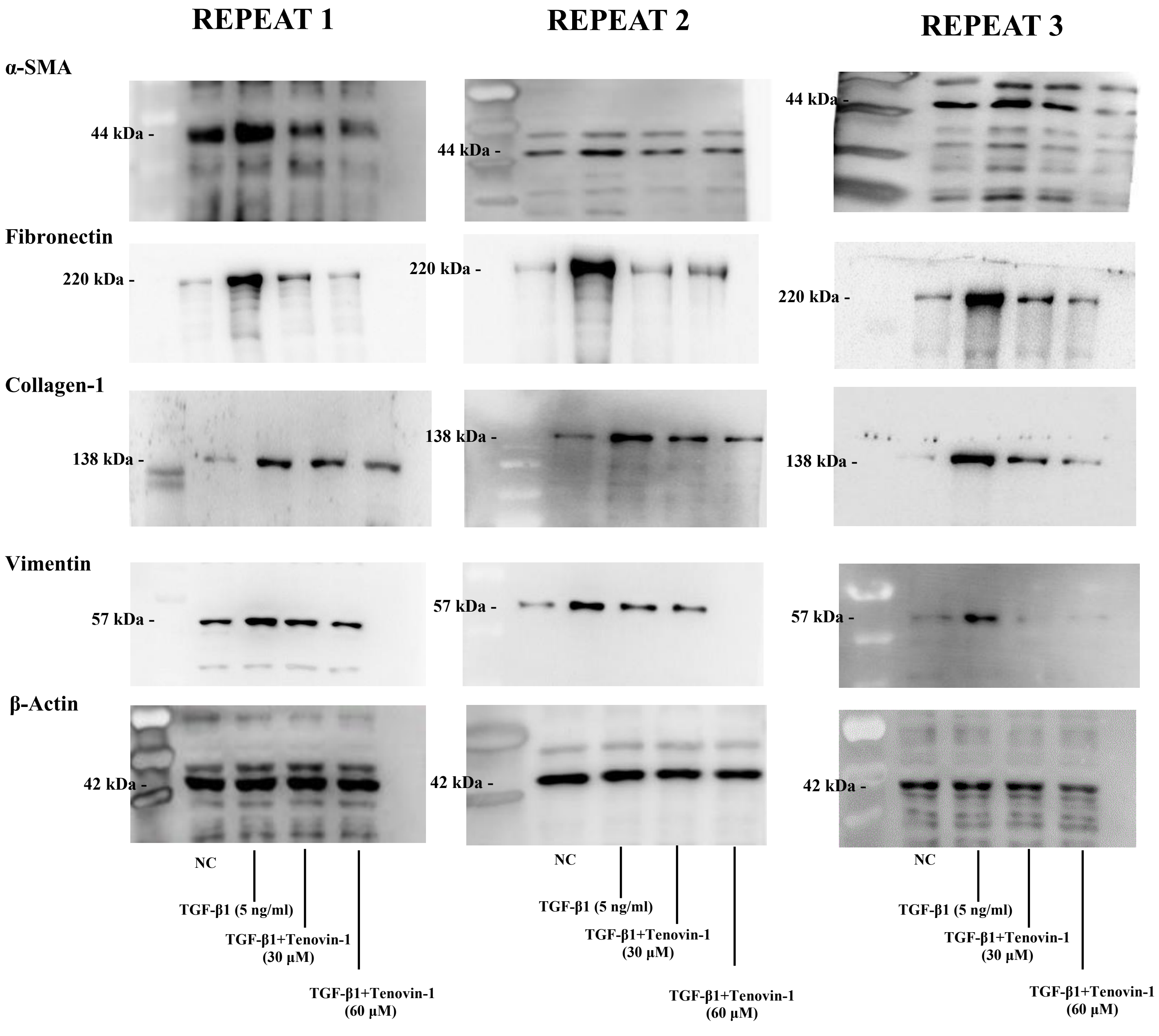

Figure S3 D: Whole blot images of **α-SMA** (44 kDa), **Fibronectin** (220 kDa), **Collagen-1** (138 kDa), **Vimentin** (67 kDa) and β-Actin (42 kDa) as represented in Figure 6B performed in 3 repeats.

E

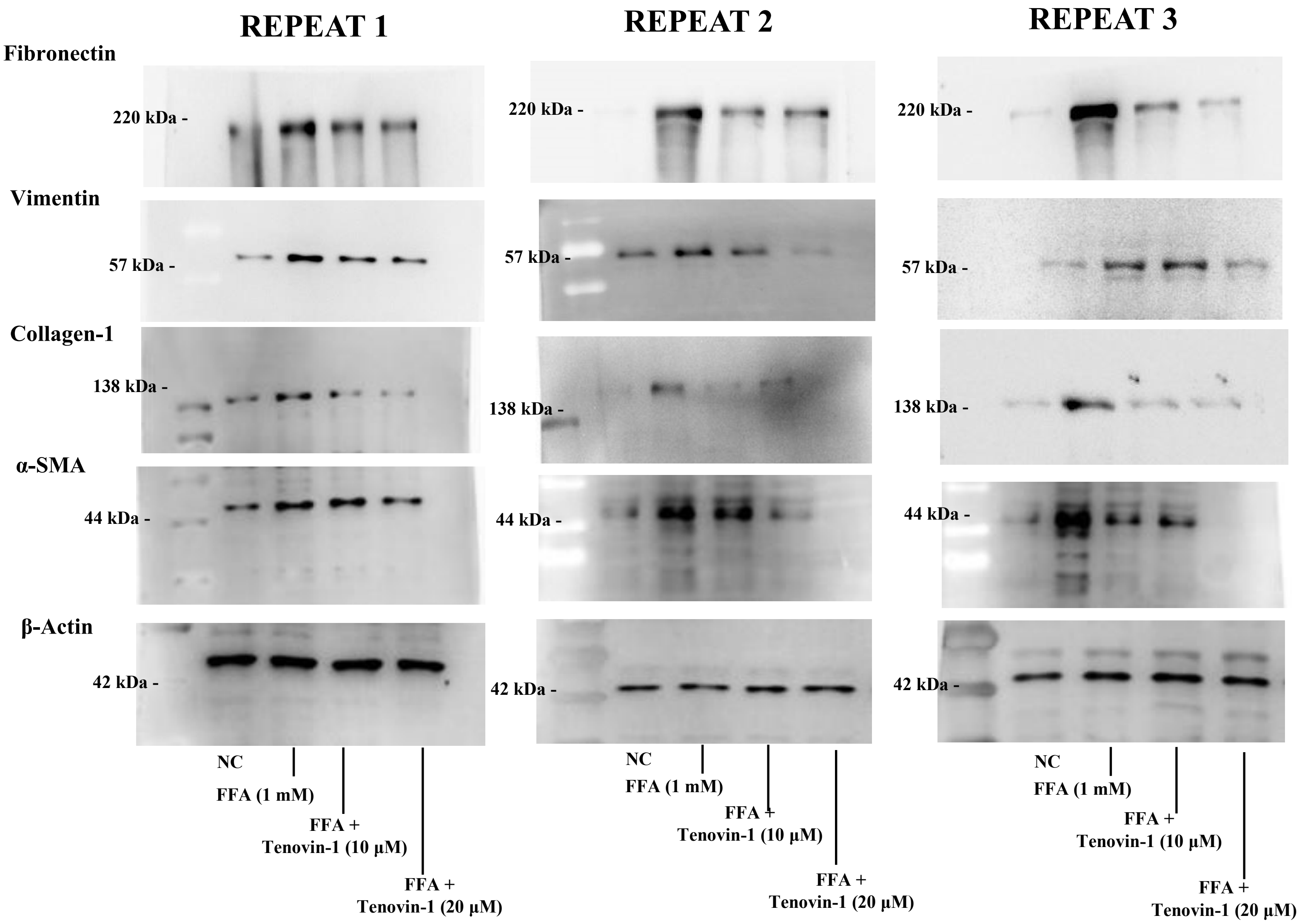

Figure S3 E: Whole blot images of **Fibronectin** (220 kDa), **Vimentin** (67 kDa), **Collagen-1** (138 kDa), **α-SMA** (44 kDa), and β-Actin (42 kDa) as represented in Figure 6J performed in 3 repeats.

F

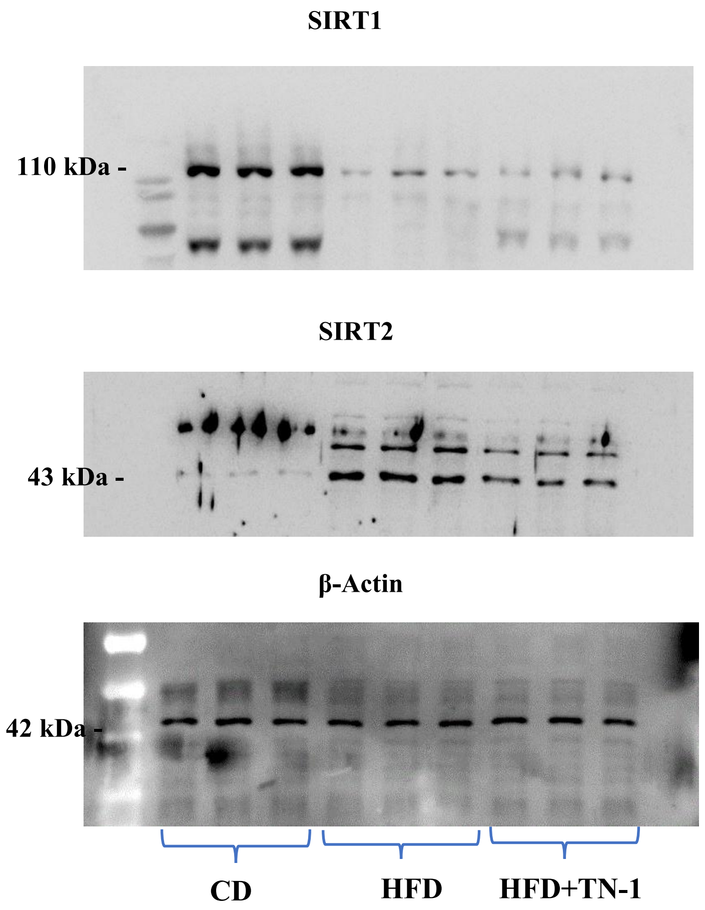

Figure S3 F: Whole blot images of SIRT1(110 kDa), SIRT2 (43 kDa), and β-Actin (42 kDa) as represented in Figure 7C.

G

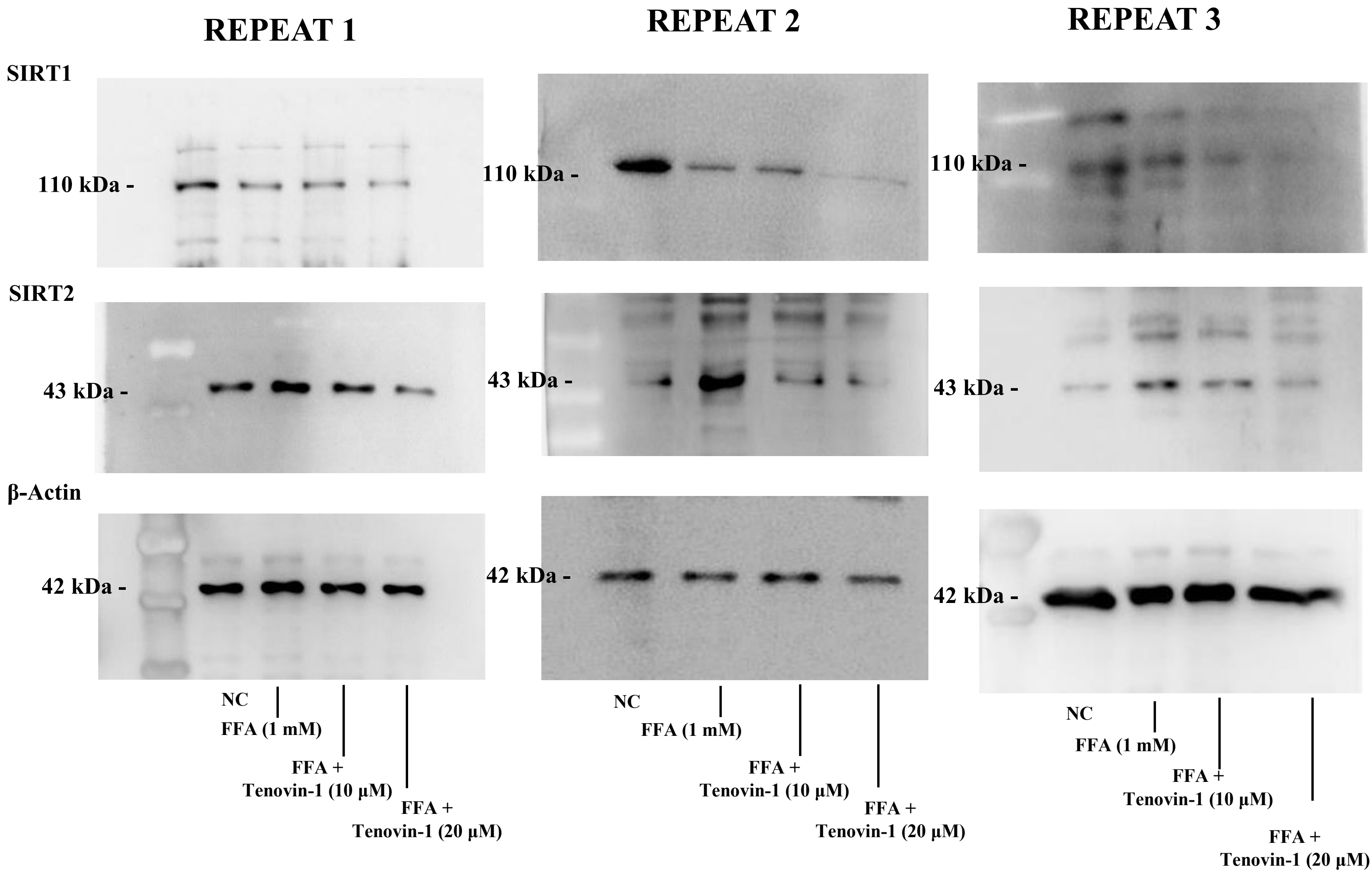

Figure S3 G: Whole blot images of SIRT1(110 kDa), SIRT2 (43 kDa), and β-Actin (42 kDa) as represented in Figure 7F performed in 3 repeats.

H

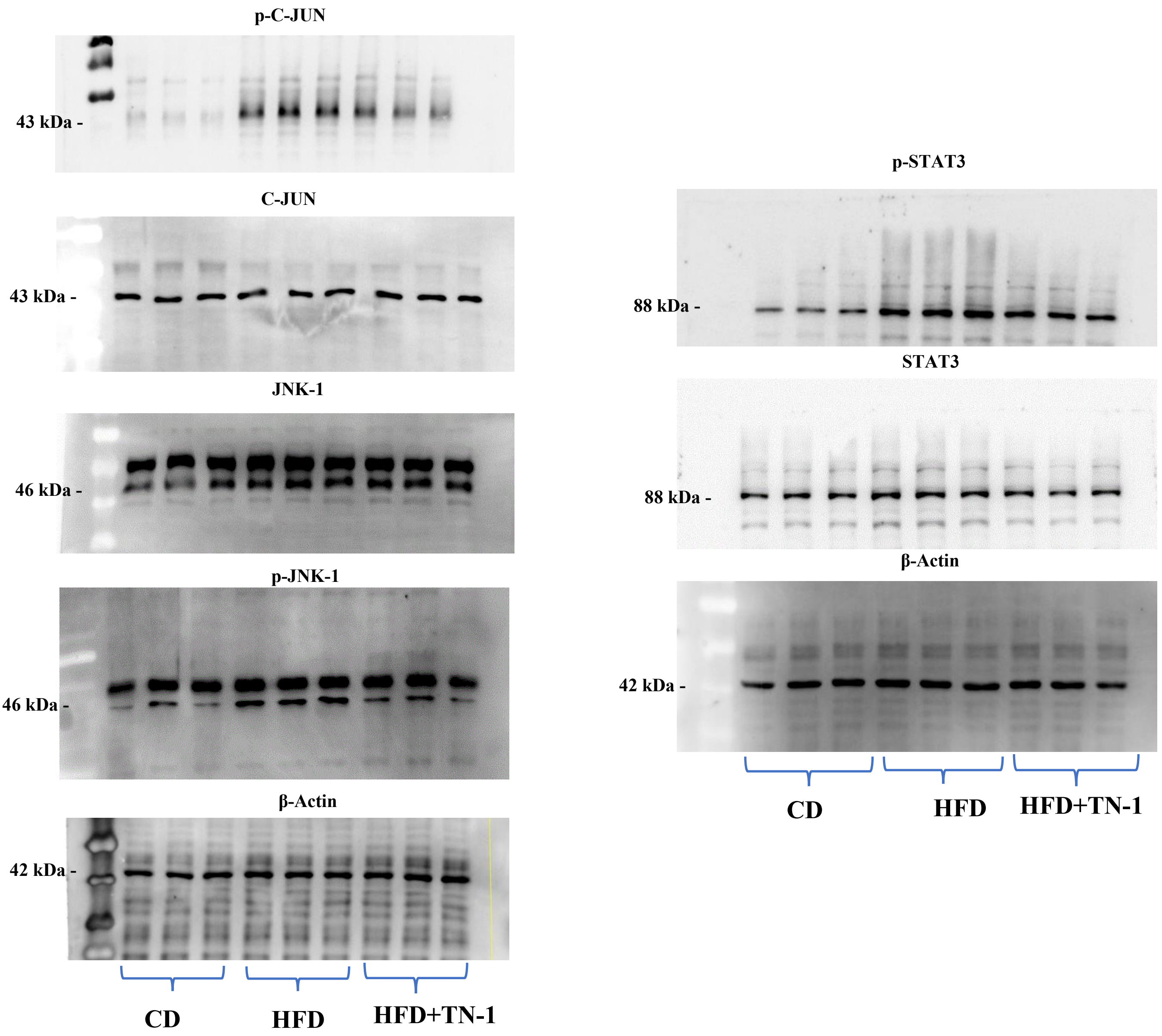

Figure S3 H: Whole blot images of p-C-JUN and C-JUN (43 kDa), JNK-1 and p-JNK (46 kDa), p-STAT3 and STAT3 (88 kDa) and β-Actin (42 kDa) as represented in Figure 8A.

I

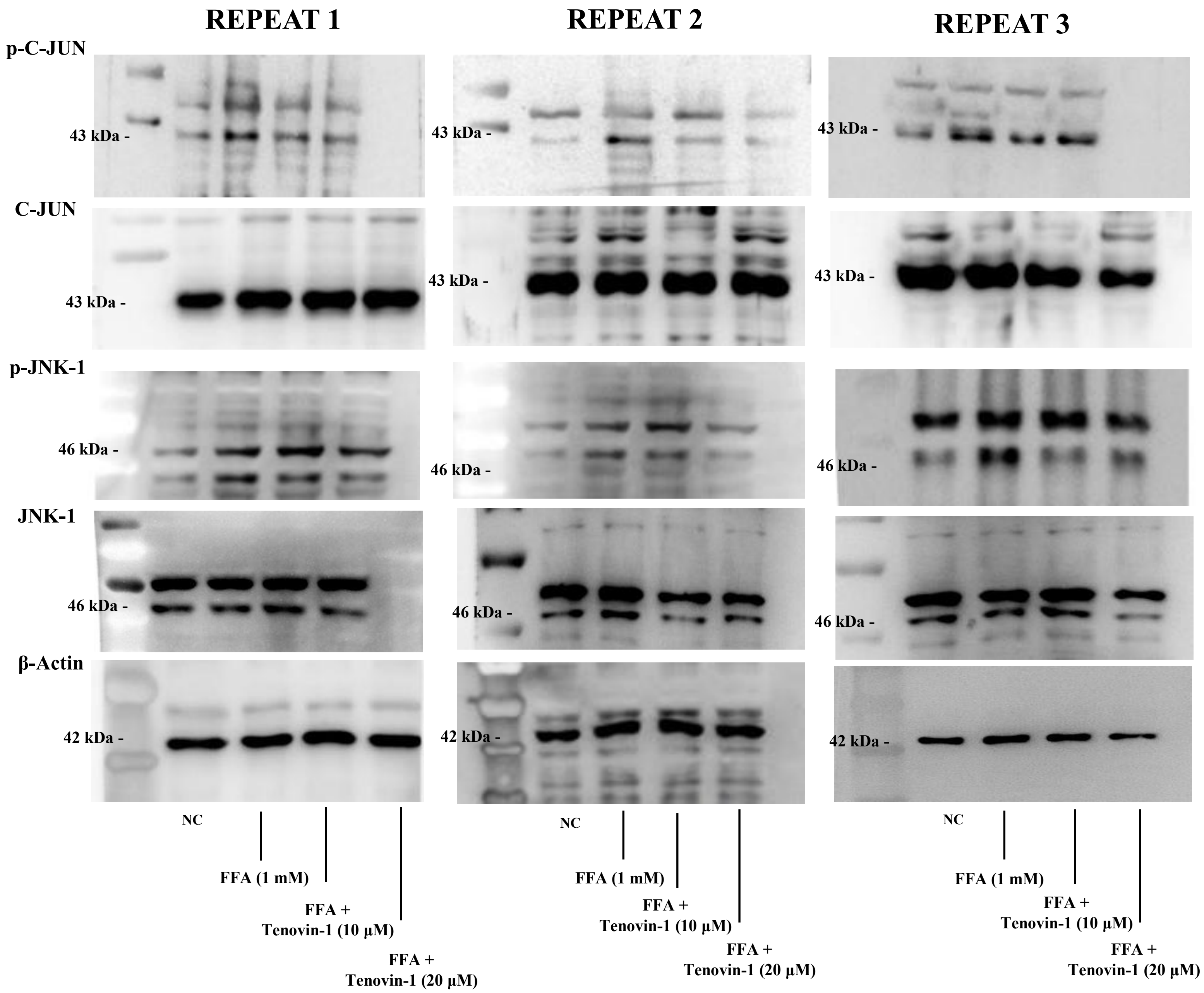

Figure S3I: Whole blot of p-C-JUN and C-JUN (43 kDa), JNK-1 and p-JNK (46 kDa), and β-Actin (42 kDa) as represented in Figure 8C performed in 3 repeats.

J

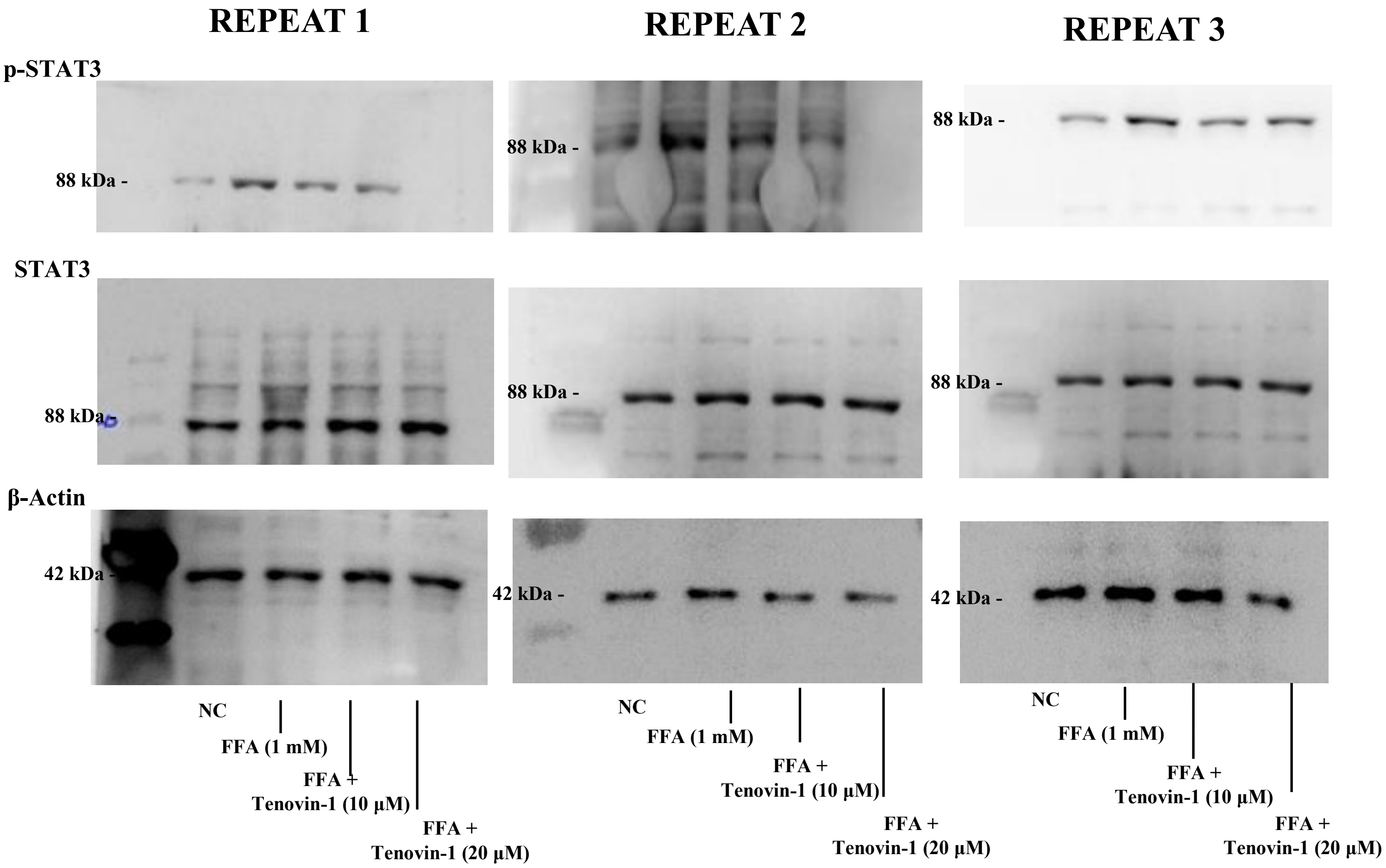

Figure S3 J: Whole blot images of p-STAT3 and STAT3 (88 kDa) and β-Actin (42 kDa) as represented in Figure 8C performed in 3 repeats.
